# High-resolution electricity generation model demonstrates suitability of high-altitude floating solar power

**DOI:** 10.1016/j.isci.2022.104394

**Published:** 2022-05-13

**Authors:** Nicholas Eyring, Noah Kittner

**Affiliations:** 1 Group for Sustainability and Technology, Department of Management, Technology, and Economics, ETH Zürich, Zürich, Switzerland; 2 Department of Environmental Sciences and Engineering, Gillings School of Global Public Health, University of North Carolina, Chapel Hill, NC, USA; 3 Department of City and Regional Planning, University of North Carolina, Chapel Hill, NC, USA

**Keywords:** Energy resources, Energy management, Energy Modeling, Energy Systems

## Abstract

This paper develops a meteorological site selection algorithm to quantify the electricity generation potential of floating solar design configurations on alpine water bodies in Switzerland. Using European power market demand patterns, we estimate the technical and economic potential of 82 prospective high-altitude floating solar sites co-located with existing Swiss hydropower. We demonstrate that the amount of solar energy radiating from high-altitude Swiss water bodies could meet total national electricity demand while significantly reducing carbon emissions and addressing seasonal supply/demand deficits. We construct a global map overlaying sites on each continent where high-altitude floating solar could provide low-carbon, land-sparing electricity. Our results present a compelling motivation to develop alpine floating solar installations. However, significant innovations are still needed to couple floating solar with existing hydropower operations or low-cost energy storage. As the industry matures, high-altitude floating solar technology could become a high-value, low-carbon electricity source.

## Highlights

• Solar energy radiating on high-altitude floating arrays could meet total Swiss demand
• Bottom-up modeling combines high-resolution meteorological data with physical model
• Site suitability tool determines electricity generation across water bodies
• All sites are economically viable before subsidies at 0.41 USD/Wp solar

## Graphical abstract

**Figure:**
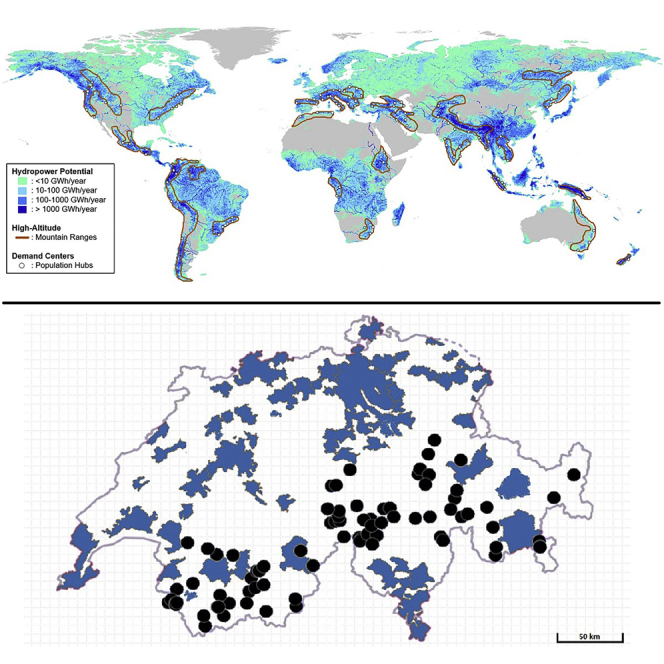

## Introduction

Global climate change requires increased urgency and attention in the energy sector to develop low-carbon electricity supply options that can dramatically reduce carbon dioxide (CO_2_) emissions (Hansen et al., 2016). Across Europe, small countries without large available land resources have developed stringent policies to decarbonize their power sectors, whereas also operating in a space where land is limited for greenfield electricity system development.

In particular, Switzerland has committed to transitioning to a clean, net-zero emissions energy system by 2050. Phasing out nuclear power will create an electricity supply gap of nearly 24.4 TWh, implying that without changes in electricity demand, countries such as Switzerland must look to alternative generation options (Swiss Federal Office of Energy, 2018). The number of choices is few – hydropower is facing financial and climate-induced risk owing to hydrologic variability and uncertainty to drought, utility-scale solar requires large land areas, distributed generation requires public buy-in and acceptance, and wind turbines are often located offshore. Therefore, high-altitude land areas could offer promising alternatives to meet carbon goals, reduce the land-use intensity of energy, and take advantage of existing electricity infrastructure, which is costly and often requires long lead-times to build. These systems can also allow existing hydropower to continue to provide flood control or other services to minimize harm from extreme hydrologic events.

**Figure 1:**
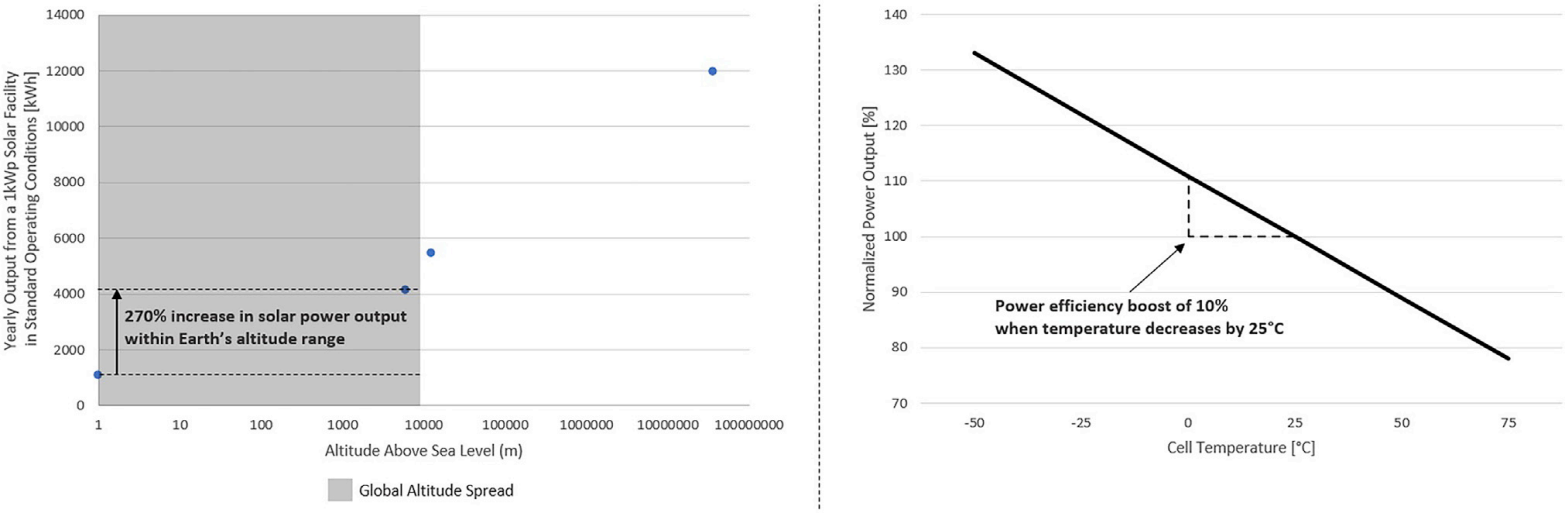
Altitude and temperature effects on solar electricity generation Left: altitude effect for annual solar power production assuming standard operating conditions. Values are taken from (Aglietti et al., 2009). Right: temperature effect on normalized power output for a current commercial solar cell. Values are taken from (Jinko Solar Team, 2021).

High-altitude solar sites generally benefit from greater electricity generation potential owing to lower radiation extinction and the high reflectance of snow (Blumthaler, 2012). Assuming standard operating conditions, the altitude effect alone can increase solar power output by 270% within Earth’s altitude range (Figure 1 – left). Solar panel efficiency also increases significantly at high altitudes owing to low temperatures (Chitturi et al., 2018), with a linear relationship between temperature decrease and efficiency boost (Dubey et al., 2013). In practice, a 10% increase in efficiency can be achieved by decreasing solar cell temperature by 25°C (Figure 1 – right). Given the land area requirement to match utility-scale solar production, the use of water bodies is a low-impact alternative to building traditional ground-mounted solar installations in mountainous terrain.

**Figure 2:**
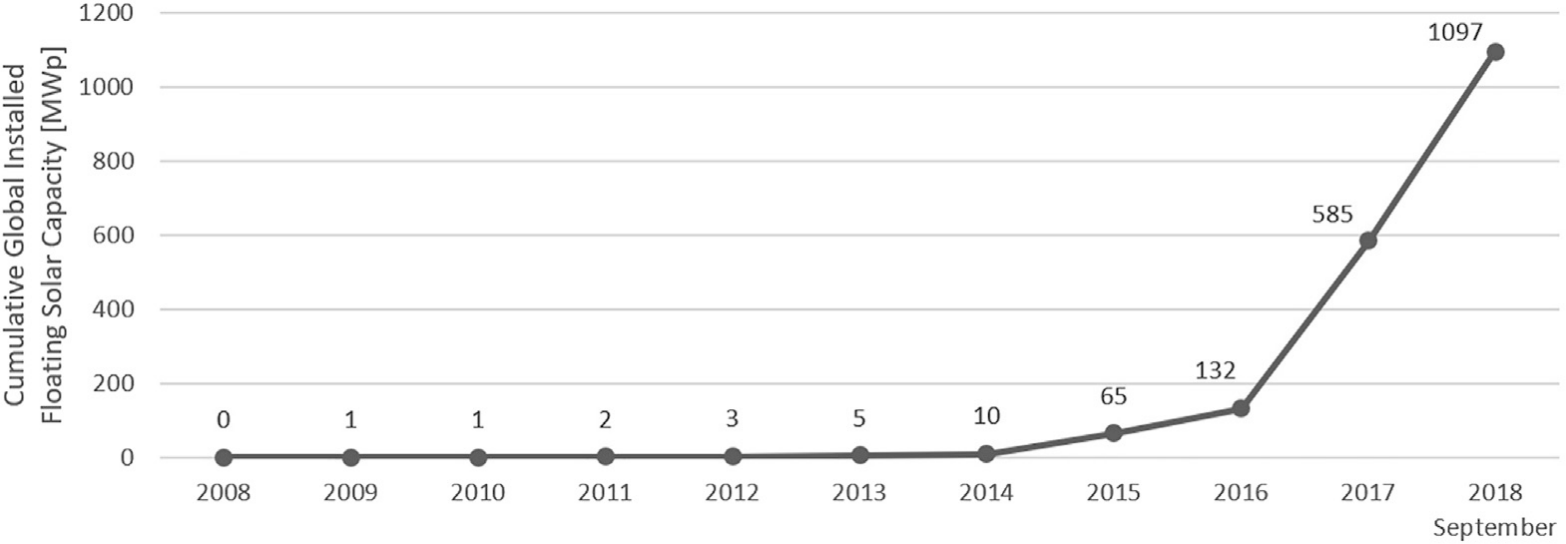
Yearly development of cumulative global installed floating solar capacity Values are taken from (World Bank Group, 2019).

Floating solar technology allows for new opportunities to increase solar capacity, especially in countries with a high opportunity cost of land (World Bank Group, 2019; Clemons et al., 2021). Floating systems boast multiple benefits compared with ground installation, including increased system efficiency owing to the natural cooling effect of water, which can decrease operating temperatures by as much as 8°C compared with an adjacent ground-mounted system (Campana et al., 2019; Sukarso and Kim, 2020). Floating arrays also diminish the need for major land preparation and allow for highly modular and reversible systems, implying less environmental impact than ground-mounted installations (Cazzaniga et al., 2019). Moreover, floating solar arrays have reduced evaporation on the surface covered by floating PV, sparing water resources (Ranjbaran et al., 2019). One study found 10% of surface water coverage would increase hydro generation as well by reducing evaporation by 70% on the covered area (Quaranta et al., 2021). Other assessments distinguish between the reduction in the evaporation rate by the type of floating solar system – with suspended systems reducing evaporation by 18%, systems fully floating on the water surface at 49%, and flexible boat models reducing the evaporation rate by 42% (Scavo et al., 2021). Costs for floating arrays are slightly higher than ground-mounted panels but are expected to decrease as production processes mature (World Bank Group, 2019). Installing solar PV systems on the downstream face of dams has also been proposed for suitability (Kougias et al., 2016a). Globally, the installed capacity of floating solar has approached exponential growth since 2012 (Figure 2), expanding from five MWp in 2013 to 1.1 GWp in September 2018 (World Bank Group, 2019). Robust floating systems capable of dealing with variable depths and harsh conditions have recently become available as standard products (Ciel and Terre International, 2019), warranting further analysis for larger scale adoption.

Consistently providing renewable electricity to satisfy variable demand remains a major technological and behavioral challenge (Davis et al., 2018). Switzerland already faces a significant temporal mismatch between demand and supply with a large winter electricity supply deficit. Current research indicates that Swiss electricity demand can fully be addressed by substituting nuclear output with a solar or low-carbon electricity-dominated portfolio. Land-use planning and access to new affordable real estate have been identified as key barriers to the required large-scale increase in solar capacity that may come from utility-scale solar (Bartlett et al., 2018). Furthermore, Swiss solar power production is typically high in summer when demand is low and insufficient in winter when electricity is most needed, with recent findings showing that mountain installations combined with higher tilt angles are suitable for rectifying this mismatch (Kahl et al., 2019).

Existing dam reservoirs often store critical water supplies and floating solar panels can offer benefits to water storage. The large number of hydropower facilities in the Swiss Alps offers existing grid connections and integration infrastructure with shared inverters and substations – a key element of net-zero emissions energy systems (Davis et al., 2018; Shan et al., 2022). The potential for combined floating solar and hydropower systems is estimated at the terawatt scale globally (Lee et al., 2020), but this analysis has focused on Switzerland in particular, where high-altitude hydropower reservoirs warrant further study. To address these gaps, our study quantifies the technical and economic potential of emerging floating solar technology on Swiss high-altitude water bodies.

## Literature review

Previous research identifies temporal mismatches between producing solar electricity and demand consumption in Switzerland (Bartlett et al., 2018). This study provides a methodology to calculate the potential electricity generation from high-altitude floating solar sites, based on geographical characteristics and panel attitude. To date, no study has evaluated how the electricity produced from floating solar PV can be incorporated with Swiss electricity supply and demand patterns and the impact on seasonal mismatches. This study evaluates the extent to which high-altitude floating solar resolves seasonal mismatches in supply and demand. Recent studies demonstrate the considerable potential of solar installation in the Swiss alps; however, these insights have not been applied to floating solar cases (Kahl et al., 2019). This study addresses this gap and applies these insights to floating solar. Many mountainous stretches remain difficult to reach, making it challenging to exploit such solar resources – thus our new research provides a feasibility test to determine whether existing dam reservoirs and transmission system interconnections are accessible for construction. Previous work has not evaluated the potential along water bodies, and has only considered land-based PV systems. In this study, we also develop a bottom-up approach to determine electricity generation potential that can be applied in other countries with high altitudes and existing hydropower dam reservoirs. Previously, these infrastructure systems have not been systematically globally evaluated for potential inclusion. These efforts will bridge a knowledge gap and provide new methods for studies across other countries seeking to mitigate seasonal electricity supply and demand mismatch challenges. This paper contributes to a new body of knowledge about the effects of altitude on floating solar generation potential. This research makes both an applied and methodological contribution to the body of knowledge on floating solar PV technology – its generation potential, application, and economic viability.

Other studies have evaluated the interplay between solar, wind, and pumped hydropower storage for Switzerland and noted the value of existing hydropower resources for power grid balancing (Dujardin et al., 2017; Kittner et al., 2021). In addition, further work explores the correlation between high-altitude solar and typical electricity demand patterns (Kahl et al., 2019). This study synthesizes the concepts of the technological interplay and complementarities arising from mountain-based solar and existing hydropower reservoirs that serve as storage or generation. Standalone and hybrid solar-hydropower storage systems have been evaluated for their optimal sizes (Xu et al., 2020; Li et al., 2018). Previously, most studies that evaluated the feasibility or complementarity of hybrid solar PV/pumped hydropower storage have done so for very small-scale systems (Jurasz et al., 2018a, 2018b; Kittner et al., 2016; Kougias et al., 2016b). However, in this paper we want to test whether there is potential – both technically and economically viable sites to increase solar generation by utilizing high-altitude mountainous reservoir sites. Previous studies identify that solar may be limited in contributing to a hybrid system – however, that could be more a function of the timing of the resource than the resource itself (Kahl et al., 2019). In this paper, we also match the generation profile of solar with typical Swiss electricity demand to estimate not only solar power output, but also timing in an approach that can be replicated for other countries and world regions. This would be a highly valuable knowledge gap for countries who are considering a phase-out of traditional electricity resources such as large-scale nuclear, coal, or hydropower and need to replace reliable electricity with a more stable resource than standalone ground-mounted solar. Our study adds value by developing a bottom-up approach to estimate solar electricity generation using a physical model that incorporates high-resolution meteorological data and analyzes the economic prospects of such a venture to play a significant role in power generation. As a result, we find that large-scale high-altitude floating solar power can significantly contribute to solving Switzerland’s capacity expansion problem – with numerous similar potential applications worldwide.

## Materials and methods

Our analysis assesses both the technical and economic potential of high-altitude floating solar technology by developing a bottom-up modeling tool that combines high-resolution meteorological data with a physical solar model to determine electricity generation across different water bodies. Solar power is intermittent by nature and can vary significantly even over short periods of time – not only owing to day/night cycles, but also to varying meteorological conditions such as cloud cover and the presence of snow (Kahl et al., 2019). National electricity production and consumption also fluctuate greatly over the course of any given day (Swissgrid, 2019). We generate expected electricity production profiles in 30-min resolution. A sample of 82 high-altitude water bodies in the Swiss alps is examined – serving as a case study with applicable results for water bodies with similar geographic properties. Key datasets were sourced from the EUMETSAT Satellite Application Facility on Climate Modeling (CMSAF) (Karlsson et al., 2019; Pfeifroth et al., 2019), and the European Network of Transmission System Operators for Electricity (ENTSO-E) (ENTSO-E Transparency Platform, 2019). To establish our sample of potential floating solar sites, Swiss water body data was sourced directly from the *Swiss Federal Office of Topography swisstopo* (swisstopo) via their interactive map of official survey and geological datasets (Swisstopo, 2019), whereas the associated Swiss hydropower plant data was retrieved from the yearly hydro statistics report published by the *Swiss Federal Office of Energy* (https://www.bfe.admin.ch/bfe/en/home/supply/statistics-and-geodata/energy-statistics.html). To calculate historic generation profiles, the solar position was computed via *Pysolar* – a python implementation of the Solar Position Algorithm (Pysolar Development Team, 2019) – with the rest of our high-resolution climate data being provided by the *EUMETSAT Satellite Application Facility on Climate Monitoring* (CMSAF) (Karlsson et al., 2019; Pfeifroth et al., 2019). To analyze the Swiss electricity supply/demand mismatch, high-resolution data on total Swiss electricity consumption and production was retrieved from *Swissgrid*, the Swiss transmission system operator (Swissgrid, 2019). For our revenue analysis, Swiss electricity price data was sourced from the *European Network of Transmission System Operators for Electricity* (ENTSO-E) (ENTSO-E Transparency Platform, 2019). Finally, Swiss grid carbon intensity data for our CO_2_-offset analysis was retrieved from an *ETH Zürich* study distributed by the *Swiss Federal Laboratories for Materials Science and Technology* (EMPA) (Chevrier et al., 2019). Further documentation can be found in the *SI Appendix*.

### Model implementation – HASPR research environment

**Figure 3:**
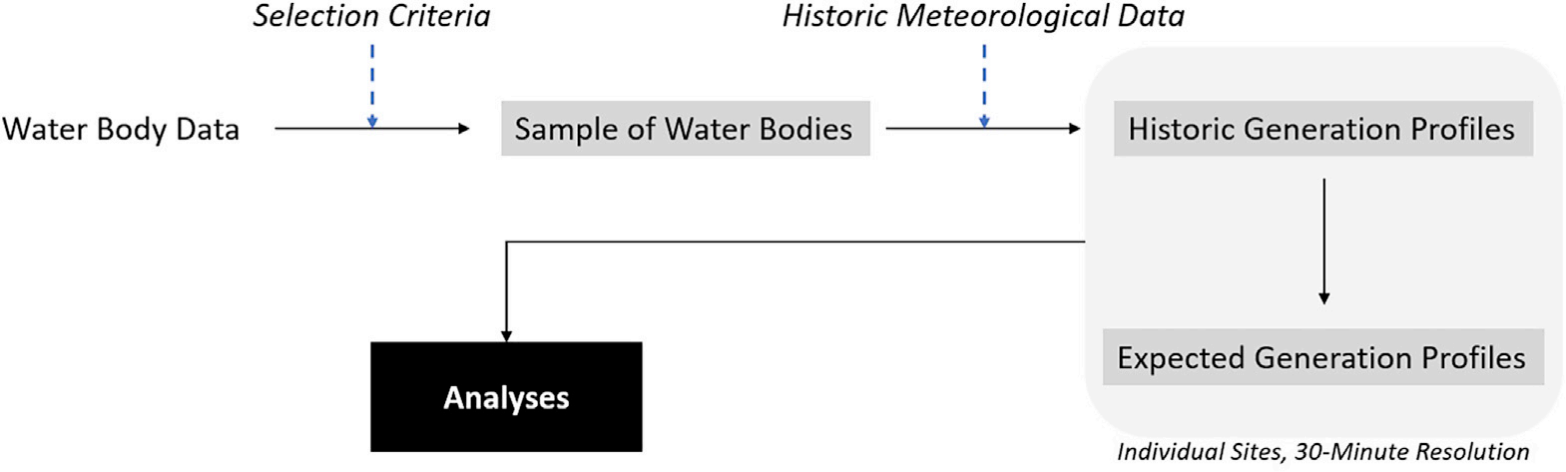
Overview of our methodology based on generation profiles of individual sites

Our analysis is based on a combination of water body data and meteorological data from which historic generation profiles are obtained in 30-min resolution for conceivable floating solar sites. Historic generation profiles subsequently serve as input for further analyses as presented in Figure 3.

We first screen water body data that fulfill our selection criteria using WGS84 coordinates, altitude, surface area, and minimum distance between the shore and official road. The database includes hydropower information such as coordinates, associated water bodies, plant type, operational status, year built, installed turbine power, installed pump power, average energy production, and storage levels. The database also consists of grid connection data such as power line location and distance to the nearest substation.

The screened sites are then combined with meteorological data such as surface incoming shortwave irradiance (W/m^2^) and surface incoming direct irradiance (W/m^2^) at a 30-min resolution, surface albedo (%) at a 5-day resolution, and solar position (altitude, azimuth). All meteorological data comes from CMSAF (Karlsson et al., 2019; Pfeifroth et al., 2019), but our implementation can utilize other data sources as long as the user inputs 30-min surface incoming shortwave and direct irradiance along with 5-day surface albedo data.

We created the High-Altitude Solar Power Research python suite (HASPR) to implement the models described herein. HASPR operates in two parts. The first part calculates electricity generation profiles for sets of latitude and longitude coordinates at a temporal resolution of 30-min. This is computationally expensive, especially when optimizing panel tilt angle and azimuth across different locations and weather inputs. Therefore, HASPR is designed to perform this step using a high-performance computer. The second part of the suite is designed to be executed on a typical local machine and consists of scripts to run analytics on generation profile data. HASPR grants users the ability to model the output of solar arrays given high-resolution meteorological data and the coordinates of sites of interest. Documentation of the code and its use can be found in the STAR Methods section (HASPR python suite – Readme).

### Systematic water body selection

Building industrial-scale power facilities in the mountains can be very expensive and difficult. To obtain a conservative lower bound for the potential of utility-scale high-altitude floating solar power in Switzerland, we focus on lakes with existing road access and nearby power infrastructure. This implies significantly lower construction costs, especially if the system’s output can be connected to the low-voltage side of existing grid-scale transformers, thereby eliminating the need to construct utility-grade transformers and surge-protection systems. Owing to the variability of solar power, coupling floating solar sites with a storage system such as pumped hydro is crucial (Ranjbaran et al., 2019). Given the large number of dams and storage hydro plants in the Swiss mountains, we selected sites associated with existing hydropower installations to obtain a sample of water bodies with the characteristics outlined above. 1,000 m is used as a high-altitude threshold. This is in line with data from the Cloudnet project’s measurements at Chilbolton Observatory, U.K. depicted in (Aglietti et al., 2009), which suggests that the majority of extinction under actual atmospheric conditions occurs below 1,000 m of altitude above sea level. Our sample encompasses all dammed water bodies in Switzerland above this threshold which are associated with storage or pumped-storage hydro facilities. Since we are estimating the potential for utility-scale installations, water bodies with surface areas less than 1,000 m^2^ are excluded (Swiss average irradiance per square meter above 1,000 m indicates that their contributions would not be significant enough to warrant a utility-scale site’s installation and maintenance costs). This exclusion also limits our sample, allowing us to better express a conservative baseline for the technology. Only dammed water bodies are considered since it is likely that disturbing pristine natural lakes would face heavier barriers to construction than building on artificial water bodies. As a result of these filters, our sample allows us to obtain a realistic lower bound to conservatively estimate the potential of floating solar power in the Swiss Alps.

The explicit criteria for adding a water body to our list of potential Swiss high-altitude floating solar sites are presented below:

*Criterion 1*: The water body is entirely in Switzerland.
*Criterion 2*: The water body is associated with a storage or pumped-storage hydro facility.
*Criterion 3*: The water body altitude is greater than 1,000 m.
*Criterion 4*: The surface area of the water body is greater than 1,000 m^2^.
*Criterion 5*: The water body is dammed.

The Swiss hydro statistics dataset we use provides us with a list of all hydro installations with a capacity above 300 kW – complete with coordinates, plant types, and associated water bodies (Swiss Federal Office of Energy, 2019). Systematically processing each data point in the hydro statistics dataset, the associated water bodies were added to our list of potential sites if all five criteria were met. Criterion 2 was automatically fulfilled given our search method. The model assumes the existing hydro facility could support extra generation. Typically, floating solar PV stations would operate at times when hydropower is not necessarily running, so the likely output would not exceed an existing facility. Once a water body associated with a storage or pumped-storage hydro facility had been identified, criterion 1 and criterion 3 were tested by reading directly from swisstopo’s interactive map (Swisstopo, 2019). Surface area data were acquired by using the map’s *VECTOR25 Primary surfaces* overlay, whereas the location of dams was determined by overlaying the *dams under federal supervision* dataset. Site coordinates were collected by right-clicking roughly in the geometric center of the water body to display point information. A rough estimate of the lake’s center suffices as our meteorological datasets are pixelated with a spatial resolution of roughly 5 km.

The described selection process results in a sample of 82 potential sites for high-altitude floating solar power production in Switzerland. In the *SI Appendix*, a summary of our sample is presented along with a full breakdown including site IDs, names, coordinates, altitudes, surface areas, associated hydro facilities, and further attributes (Tables S3A and S3B).

**Figure 4:**
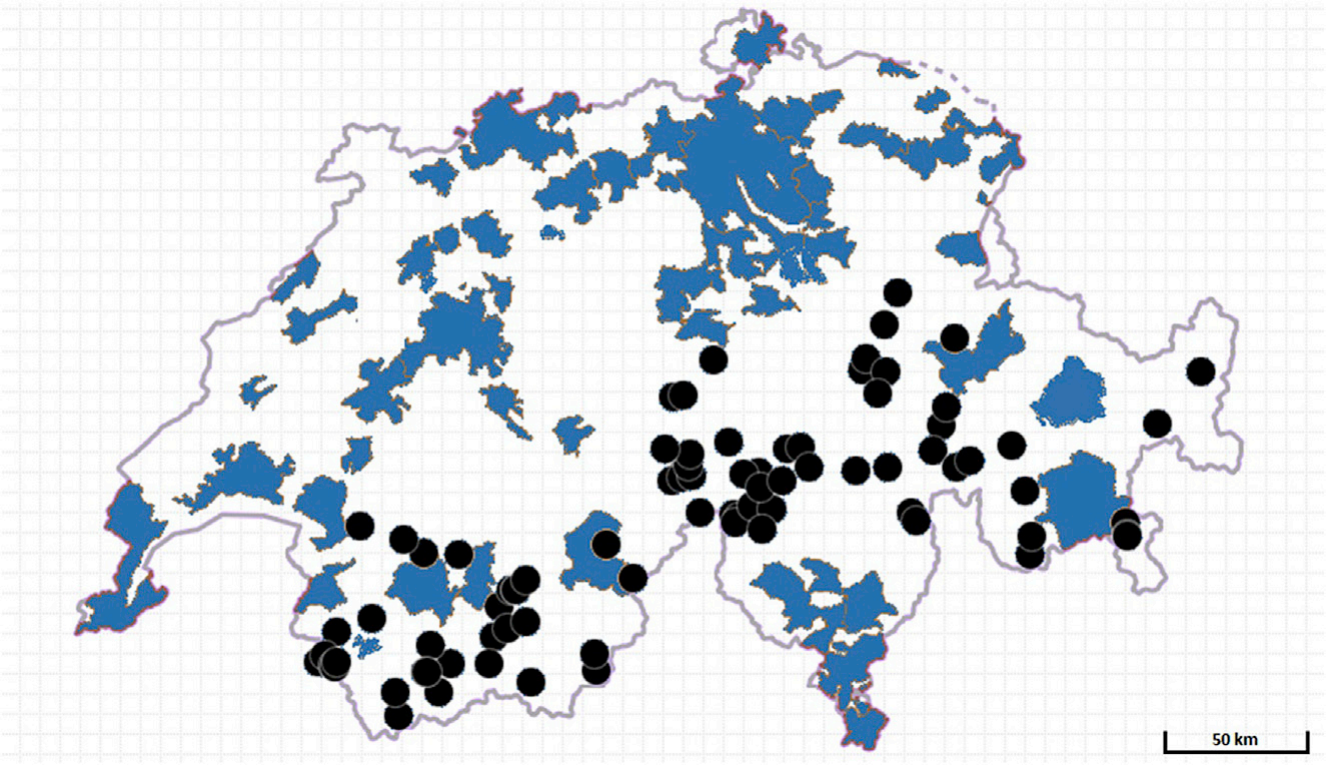
Location of the 82 water bodies in our Swiss sample (black dots) Areas shaded in blue represent agglomerations, with data taken from (Swisstopo, 2019).

Exclusively considering water bodies at altitudes above 1,000 m and with surface areas greater than 1,000 square meters, our sample consists of 82 high-altitude water bodies in Switzerland with an average surface area of 0.61 square kilometers (total surface area: 50.1 sq. km) and an average altitude of 1,783 m, representing a feasible baseline of high-altitude floating solar sites with hydropower integration options. Figure 4 presents the locations of the sites in our sample along with Swiss agglomerations to illustrate distances to electricity demand centers. Associated utility-scale hydro facilities provide grid connections (substations and 380 kV/220 kV lines) allowing for electricity distribution on a national scale. In this case, for Switzerland, we can assume that grid transmission loss is negligible.

### Technical generation potential calculated with a high-resolution plane of array (POA) model

A generation profile expresses the electricity output over time of a potential floating solar site. The primary factor in determining the output of a solar power system is the level of incoming solar radiation (Antonanzas et al., 2016). Consequently, our approach calculates expected generation profiles for each site in our sample based on the most recent 10 years of available historic radiation data. Climate data records provided by CMSAF were used owing to their high temporal resolution (30 min for radiation data) as well as their extensive validation and calibration, as described in (Pfeifroth et al., 2019).

Panel position has a significant influence on the power generated by floating solar arrays (Cazzaniga et al., 2019). Similar to ground-mounted solar plants, floating solar systems exist in a variety of design configurations ranging from fixed-position systems to solar tracking designs (Ranjbaran et al., 2019). To present and compare results for multiple system types, we calculate generation profiles for five panel position cases – ranging from flat panels to solar tracking designs.

### Modeling historic generation profiles

The HASPR suite optimizes panel tilt and azimuth angles based on latitude and longitude coordinates and weather data. The model estimates optimal generation profiles based on ten years of historical weather patterns for each given site – maximizing electricity production either for the entire year or for winter (November–April). This tool and methodology can be applied to any latitude and longitude location.

Solar energy harnessed by a panel can be broken down into three components: the energy from the direct beam (direct component), the energy from all the scattered beams in the sky (diffuse component), and finally the beams reflected from the ground (ground-reflected component) (Kahl et al., 2019; Kern and Harris, 1975). We assume that the diffuse radiation is isotropic, meaning that the scattered beams are evenly distributed over the hemisphere in question for simplicity and to make our results comparable with existing high-altitude PV studies (Kahl et al., 2019). There may be some limitations owing to anisotropy of snow reflectance with grain size, zenith angle, wavelength, and snow wetness, and further work could account for alternative transposition models such as Perez4 (Yang, 2016). Isotropic Plane of Array (POA) models are suitable for determining baseline energy production – calculating panel output by projecting multiple incoming components onto a vector that is perpendicular to the panel’s surface (Lave et al., 2015). Multiplying the resulting incoming solar energy per square meter by the system’s efficiency yields the amount of electricity generated per unit of surface area. This process is described in Equations 1 and 2, where η represents the system efficiency.

(Equation 1) $$E_{POA}=E_{direct}+E_{diffuse}+E_{ground-reflected}$$

(Equation 2) $$E_{out}=\eta \cdot E_{POA}$$

**Figure 5:**
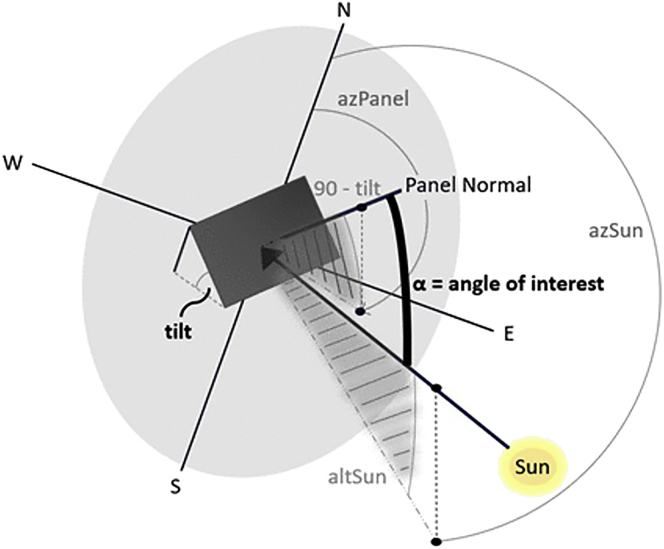
Geometric diagram to obtain projection of direct component onto panel normal vector See Equations 3 and 4

The first term in Equation 1 denotes the projection of the direct beam onto the panel normal vector. We define α as the angle between these two vectors, $\theta_{Z}$ as the solar zenith angle, and use the Surface Incoming Direct (SID) irradiance for one horizontal square meter to rewrite the direct component as shown in Equation 3. The cosine of α can be determined by transforming the current solar position and panel latitude from two points in spherical coordinates to two vectors in cartesian space. Since we are only interested in the angle, assuming both vectors have an amplitude of one allows us to determine the cosine of α via their scalar product. The result is presented in Equation 4, where γ represents solar azimuth, β is the panel tilt, and $\gamma_{P}$ denotes panel azimuth. If the sun is behind the panel (cos(α)<0), we set *E*_*direct*_ to zero. A geometric representation is shown in Figure 5.

(Equation 3) $$E_{direct}=\frac{SID}{\cos\left(\theta_{Z}\right)}\cdot \cos\left(\alpha \right)$$

(Equation 4) $$\cos\left(\alpha \right)=\sin\left(\gamma \right)\cdot \cos\left(\gamma \right)\cdot \sin\left(\beta \right)\cdot \cos\left(\gamma_{P}\right)+\sin\left(\gamma \right)\cdot \sin\left(\gamma \right)\cdot \sin\left(\beta \right)\cdot \sin\left(\gamma_{P}\right)+\cos\left(\theta_{Z}\right)\cdot \cos\left(\beta \right)$$

The second term in Equation 1 represents the projection of all scattered beams onto the panel normal vector. To express this term assuming isotropic diffuse radiation, we multiply the Surface Incoming Diffuse irradiance for one horizontal square meter (SIDIFF) by a sky view factor as described in (Hay, 1993). Equation 5 presents the result.

(Equation 5) $$E_{diffuse}=SIDIFF\cdot \left(\frac{1+\cos\left(\beta \right)}{2}\right)$$

The energy from beams reflected off the ground and nearby surfaces is represented by the third and final term in Equation 1. We assume that the reflection is isotropic, allowing us to use a ground view factor combined with the surface albedo (${\text{ρ}}_{surface}$) and Surface Incoming Shortwave irradiance for one horizontal square meter (SIS) as presented in (Hay, 1993). The resulting expression for the ground-reflected component is shown in Equation 6.

(Equation 6) $$E_{ground-reflected}=SIS\cdot \rho_{surface}\cdot \left(\frac{1-\cos\left(\beta \right)}{2}\right)$$

Although three radiation datasets are mentioned in our model’s equations, only two are necessary to collect since SIS is defined as the sum of SID and SIDIFF (Pfeifroth et al., 2019).

SIS and SID datasets were retrieved at a temporal resolution of 30 min for the years 2008–2017. However, the maximum resolution provided by CMSAF for surface albedo is 5 days with values only until the end of the year 2015. To obtain historic generation profiles in 30-min resolution, we take the average of the 5-day surface albedo over the years 2006–2015 at the coordinates in question. Solar altitude and azimuth are calculated for every time step via *Pysolar* (Pysolar Development Team, 2019) (with $\theta_{Z}$ = 90° – solar altitude) and panel position parameters were set for each case as outlined below:

Case 1: Flat panels
  • β = 0, implying that $E_{POA}=SIS$
Case 2: Tracking panels
  • At every time step, β = $\theta_{Z}$ and $\gamma_{P}$ = γ
Cases 3 & 4: Fixed panels with 12° tilt

These cases represent the standard configuration of the current floating solar market leader (Ciel and Terre International, 2019). Brute-force with an increment of 10° was used to optimize $\gamma_{P}$ with β = 12 for both winter production (November-April, Case 3) and total production (Case 4) for the year 2017, representing the most recent available radiation data.

Case 5 : Fixed panels with tilt between 30 and 65°, optimized for winter

The configuration needed to maximize winter production with high-altitude fixed panels in Switzerland entails setting the tilt between 30 and 65° (Kahl et al., 2019). For this case, we used brute-force to optimize $\gamma_{P}$ (increment = 10°) and optimized β between 30 and 65° (increment = 5°). The optimization was run for the year 2017, maximizing winter output (November-April).

Historic generation profiles were calculated for every water body in our sample, for all five panel position cases, and for all 10 years between January 1, 2008 and December 31, 2017. As a baseline, system efficiency was set to 15%, as applied in (Kahl et al., 2019). It should be noted that, given our model, power generation is linear with respect to system efficiency, allowing for simple extrapolation of our results to other panel efficiency values – for example, to identify upper bound limits as future panels increase in conversion efficiency.

### Calculating expected generation profiles

Given 10 years of historic generation output, we calculate the expected yearly generation profile per square meter for each site by averaging the historic values at every time step according to Equation 7, where ${\hat{g}}_{i,T=t}$ is the expected generation per square meter for site *i* at time step *t* and $g_{i,T=t}$ is the corresponding historic generation per square meter. For consistency, leap days are disregarded.

Owing to the intermittent nature of solar power, it is desirable to obtain insights into the variability and uncertainty of electricity production. For our analysis, we determine a lower bound for 30-min site output at 95% confidence by adding a noise term to Equation 7. To achieve this, the output is modeled as the expected value of a stochastic variable following a normal distribution centered around the average historic production and with a variance equal to the variance in historic generation for the corresponding time step as expressed in Equation 8, where $\sigma_{i,T=t}^{2}$ is the variance in historic output per square meter of site *i* at time step *t*. The noise term is used solely to determine lower bounds as its use to determine expected output could falsely add power from the tails of the distribution. Equation 8 is consistent with Equation 7 as the expected value of the normal distribution simply equals its mean.

(Equation 7) $${\hat{g}}_{i,\;T=t}=average\left(g_{i,T=t}\right)$$

(Equation 8) $${\hat{g}}_{i,\;T=t}=E\left[n_{i,\;T=t}\right]\;,\;where\;n_{i,\;T=t}∼N\left(average\left(g_{i,T=t}\right),\;\sigma_{i,T=t}^{2}\right)$$

Our implementation of these calculations produces yearly expected generation profiles in 30-min resolution along with the lower bound (95% confidence), the variance, and the normalized variance (equal to variance divided by expected output), and contribution breakdowns at each time step for direct, diffuse, and reflected irradiation.

### Aggregation of individual generation profiles

Historic and expected generation profiles compute the electricity generated at individual sites in terms of energy per square meter. Multiplying by the respective panel surface area results in the actual energy produced. To gain insight into the potential electricity production of our entire water body sample, we take the sum of the expected energy generation across all sites. This calculation is expressed in Equation 9, where *G*_*T* = *t*_ denotes the electrical energy generation across the entire sample at time step *t* and *PSA*_*i*_ is the panel surface area at site *i*. To obtain a range of results, *PSA*_*i*_ was set to various percentages of the corresponding water body’s surface area.

(Equation 9) $$G_{T=t}=\sum_{i}\left({\hat{g}}_{i,\;T=t}\cdot PSA_{i}\right)$$

### Measuring the temporal supply/demand mismatch

We measure the Swiss electricity supply/demand mismatch at a given point in time by taking the difference between total electrical energy production and total electrical energy consumption in the Swiss control block. If consumption is greater than production, the difference needs to be imported from neighboring countries. To quantify the extent to which high-altitude floating solar power can address the Swiss domestic supply/demand mismatch, we determine the amount of these imports which could be offset given aggregate expected generation profiles for each of our panel position cases under various surface coverage scenarios.

**Figure 6:**
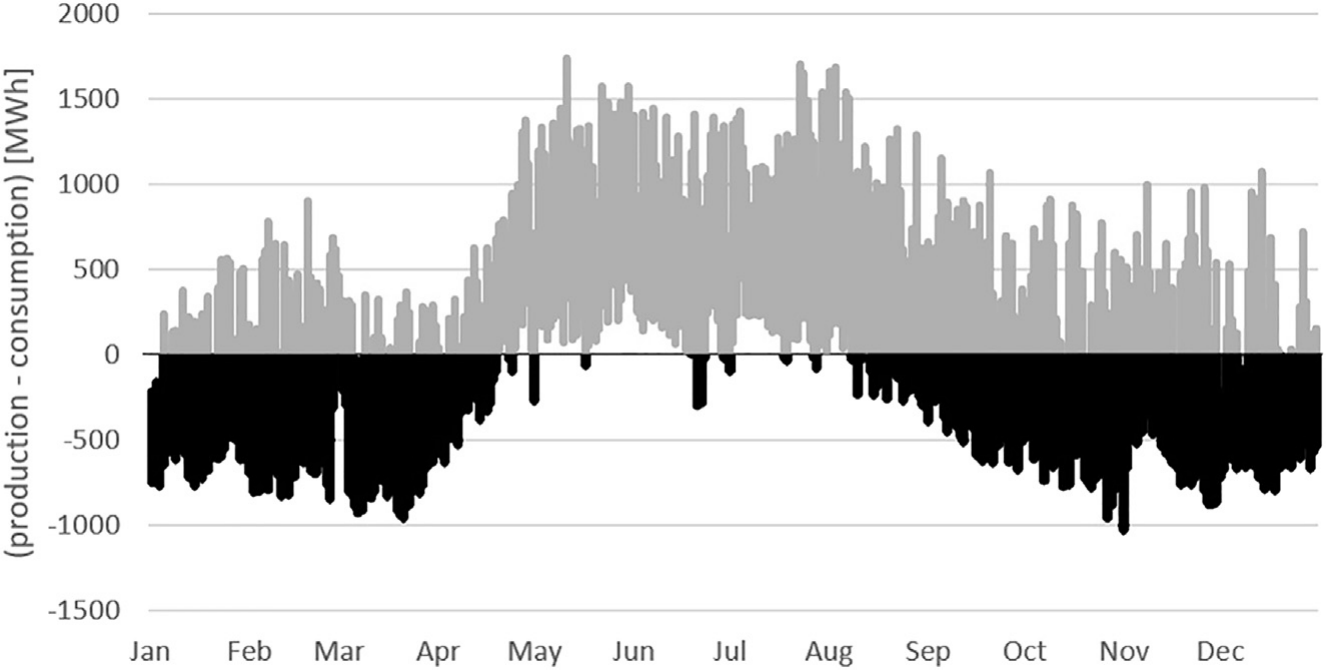
Swiss temporal mismatch between electricity supply and demand in 2018 (15-min resolution) with black data points representing insufficient domestic supply Values are based on data from (Swissgrid, 2019).

The data needed for this analysis is available in 15-min resolution from (Swissgrid, 2019), allowing us to compare the mismatch with aggregated generation profiles in 30-min resolution by summing the difference between production and consumption in half-hour steps. 2018 data is used to represent the most recent values available for an entire year. For reference, the resulting mismatch between Swiss electricity consumption and production is presented in Figure 6. During the summer, excess electricity from floating solar can be sold abroad into European electricity markets if there is surplus generation.

### Measuring CO_2_ offset potential

To gain insight into the positive environmental effects of installing high-altitude floating solar power in Switzerland, we estimate the amount of CO_2_-equivalent greenhouse gas emissions that could be offset for various aggregate generation profiles. Given the hourly intensity of CO_2_-equivalent emissions for the Swiss electrical grid from Chevrier et al. (2019), we multiply the emission values per unit of energy by the hourly floating solar output to obtain the CO_2_-equivalent offset if floating solar power is used as a substitute for current non-zero emissions energy sources – assuming the power is sold at the time of generation. To put the results into perspective, we compare the offset with annual European CO_2_ emissions from coal power with data provided in U.S. Energy Information Administration (2019).

### Revenue analysis via the Swiss wholesale market

Our bottom-up analysis of the market potential of high-altitude floating solar in Switzerland requires us to compute the potential revenue of each site in our sample. To determine the revenue potential without government intervention, subsidies and feed-in tariffs are not considered. Instead, we calculate the corresponding revenue profile for a given generation profile by assuming that power is sold at the time of production on the Swiss wholesale market, for which hourly prices are provided for the years 2015–2018 in ENTSO-E Transparency Platform (2019). To sell power in this market, bids must be defined for hourly slots in increments of 0.1 MWh (Abrell, 2019). We therefore round the generation over a given slot down to the nearest 0.1 MWh to determine bid revenue. Equation 10 expresses the revenue calculation for one site, where *B*_*i*_ represents the bid revenue for site *i* over the year in question and *price*_*t*_ denotes the slot price at time *t*. Values for expected revenue were calculated by averaging the results over the period 2015–2018.

(Equation 10) $$B_{i}=\sum_{t\in year}\left(round\_down\left(\sum_{u\in \left[t,t+1h\right]}{\hat{g}}_{i,\;T=u}\cdot PSA_{i},\;0.1MWh\right)\cdot price_{t}\right)$$

For the sake of analysis, our implementation of the revenue calculation also outputs the total unsold power for each slot in addition to the total potential revenue if all generated power was sold – for example through the coupling of floating solar output with a non-intermittent electricity source.

### Estimating site costs

Site costs are modeled as the sum of upfront construction costs (capital costs) and a yearly Operations and Maintenance (O&M) cost equal to a percentage of the initial investment. The resulting cumulative cost is expressed in Equation 11, where *CC* represents the capital costs, *OM%* denotes the O&M percentage, and *L* is the lifetime of the system in years.

(Equation 11) $$cumulative\_cost_{year\;y}=CC\cdot \left(1+y\cdot OM\%\right),\;for\;y=1\;to\;L$$

Three sources were used to estimate the current capital costs of utility-scale floating solar sites in Switzerland: the most recent World Bank report on the global floating solar market (World Bank Group, 2019), Campana et al. (2019) on the topic of optimizing and assessing floating solar systems, and a 2018 study on the use of floating solar plants in coordination with hydropower (Silvério et al., 2018). System costs are expressed per watt-peak (Wp), which denotes the output of a site under standard test conditions – defined at 25°C with an air mass coefficient of 1.5 and where the total incoming radiation on the panel equals 1,000 W per square meter (Er et al., 2018). For simplicity, we assume a standard system efficiency of 15%, resulting in 150 Wp per square meter. Multiplying this value by the panel surface area of the respective site yields the power rating in Wp from which we determine capital costs. The *SI Appendix* contains a summary of the values retrieved from our three sources and their conversion to CHF/Wp (Tables S5 and S6). We average these values to establish a capital cost of 1.43 CHF/Wp for floating solar arrays with flat panels (Case 1).

Capital costs rise for floating platforms and anchoring systems as panel tilt increases (Silvério et al., 2018). Therefore, to determine the upfront costs for Cases 3 and 4 (fixed tilt at 12°), we calculate the marginal increase in cost per degree of tilt through a linear regression on data presented in (Silvério et al., 2018) – resulting in an increase of 0.0187 CHF/Wp for each degree (*R*^2^ = 0.98) and a total capital cost of 1.65 CHF/Wp for these two cases.

Owing to harsh weather conditions in the Swiss alps, Cases 2 and 5 represent hypothetical systems for which no standard products exist, but we expect these configurations are feasible. We therefore exclude these cases from our bottom-up costs and investment profiles analyses and instead present their revenue and generation profiles as a motivation for further research and development, along with hypothetical investment profiles if these systems would be built at the same costs as 12° panels.

To establish a baseline for the cost profiles of individual sites, yearly O&M costs were set to 2% of capital costs as is the case in the floating solar cost analysis presented in (Campana et al., 2019). This is a reasonable and conservative measure based on reviews of other existing floating solar installations (Gorjian et al., 2020; Rosa-Clot and Tina, 2020; Spencer et al., 2018). Spencer et al. (2018) document distinct cost advantages of leveraging existing transmission infrastructure for combined hydropower and floating solar power plants. The Longyangxia plant in Qinghai, China is an 850-MW floating solar PV plant on a 1,280-MW hydropower reservoir with no solar curtailment and smooth generator output (Spencer et al., 2018), denoting cost advantages unmet by ground-mounted utility-scale solar systems.

### Economic viability calculations – Levelized cost of electricity (LCOE) and net present value (NPV)

For all sites in our sample, we establish investment profiles by calculating the LCOE and the NPV under each of our investigated design configurations. LCOE was computed according to Equation 12, from Darling et al. (2011), whereas Equation 13 describes our calculation of NPV. Descriptions of the relevant terms and parameters can be found in Table 1.

(Equation 12) $$LCOE=\frac{CC\;+\;\sum_{n=1}^{L}\left(\frac{AO}{{\left(1+DR\right)}^{n}}\;-\;\frac{RV}{{\left(1+DR\right)}^{n}}\right)}{\sum_{n=1}^{L}\frac{IP\cdot {\left(1-SDR\right)}^{n}}{{\left(1+DR\right)}^{n}}}$$

(Equation 13) $$NPV=-CC+\sum_{n=1}^{L}\frac{CF_{n}}{{\left(1+DR\right)}^{n}}+\frac{RV}{{\left(1+DR\right)}^{L}}$$

**Table 1:** Description of terms and parameters used in LCOE and NPV calculations

Team/Parameter	Description	Value Used
*CC*	Capital costs	[see corresponding section]
*L*	System lifetime in years	25
*AO*	Annual operations cost	2% of capital costs
*DR*	Discount rate	7%, 8%, 10%
*RV*	Residual value	10% of capital costs
*IP*	Initial production	Expected yearly generation
*SDR*	System degradation rate	0.5%
*CFn*	Cash flow in year n	Expected yearly revenue∗(1-SDR)ˆn-AO

Our analysis assumes a system lifetime of 25 years, based on current available technology and typical values found in solar power literature (Khiareddine et al., 2018; Quaranta et al., 2021). To set the system degradation rate, we use results from a 2018 paper stating that reliability studies on floating solar technology have demonstrated rates below half a percent per year for performance loss (Kamuyu et al., 2018). Therefore, we assume a yearly degradation of 0.5% to conservatively estimate lifetime generation and revenue.

Finally, we assume that the residual value of a floating solar site is equal to 10% of the initial project cost, as is the case in the analysis presented in Silvério et al. (2018). Given the investment profiles for individual sites, aggregate profiles for each design configuration were determined by averaging LCOE and summing NPV, respectively.

### Model limitations

Generation profiles have been validated by comparing the average annual output to published results. Our sample’s yearly average of 133 W/m^2^ is consistent with data presented in (Kahl et al., 2019) and confirms this study’s conservative approach. However, the primary limitation of our POA model lies in the spatial resolution of the meteorological datasets. At roughly 5 km, the pixel resolution is too low to take topographic shading into account for many of the water bodies, potentially distorting output results. Furthermore, the model’s assumptions of isotropic diffuse radiation and constant system efficiency (assuming no panel snow cover and temperature effects) limit the precision of the values presented herein. The SARAH data product used in the study does not explicitly discriminate between clouds and snow, which can underestimate irradiance in the winter. For a first-cut analysis, the differentiation would not dramatically alter the results, as one can see the majority of the electricity supply gap in Switzerland occurs in winter months (Figure 6). In addition, our models do not account for the accumulation of snow on floating panels. Instead, in this initial analysis, we present results that assume that snow cover is dealt with through operations and maintenance or that snow will slide off panels with high tilts. Further, ground-reflected solar irradiance is drastically reduced when panels are mounted in multiple rows. This model assumes an undisturbed view onto a flat ground with the albedo of the surrounding terrain. Finally, the future development of electricity prices has not been considered in this study. If prices fall, high-altitude floating solar may not be economically viable in Switzerland even if the cost targets we presented are achieved.

## Results

Individual results for each site depicted in Figure 4 have been established at 15% efficiency at different levels of surface area coverage. The *SI Appendix* includes high-resolution individual profiles and links to supporting documents (Table S1, HASPR readme). Supporting documents contain additional information for each water body, including site topography and locations of associated hydropower facilities (Tables S3A and S3B). Tables S7 and S8 detail the annual output for different water bodies.

### National-scale technical potential

**Figure 7:**
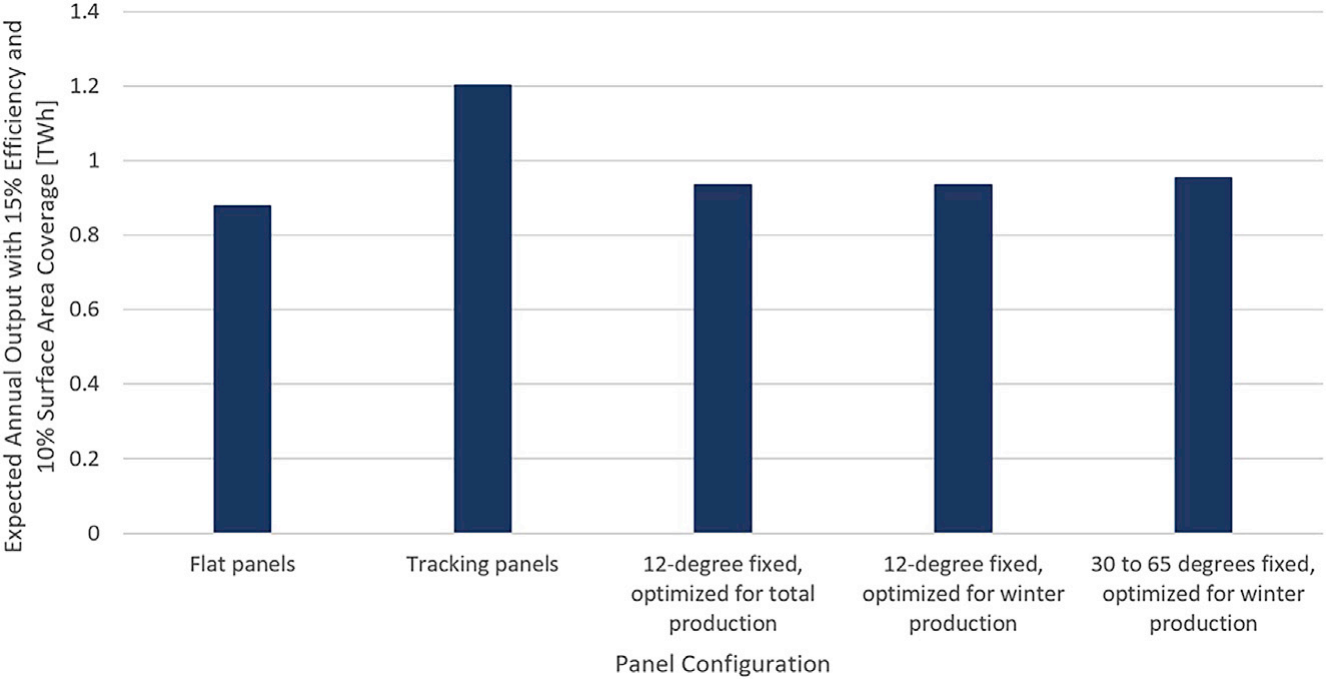
Expected annual output across all 82 water bodies in our sample under various panel configurations Values assume 10% of the surface area is covered by panels operating at 15% efficiency.

**Figure 8:**
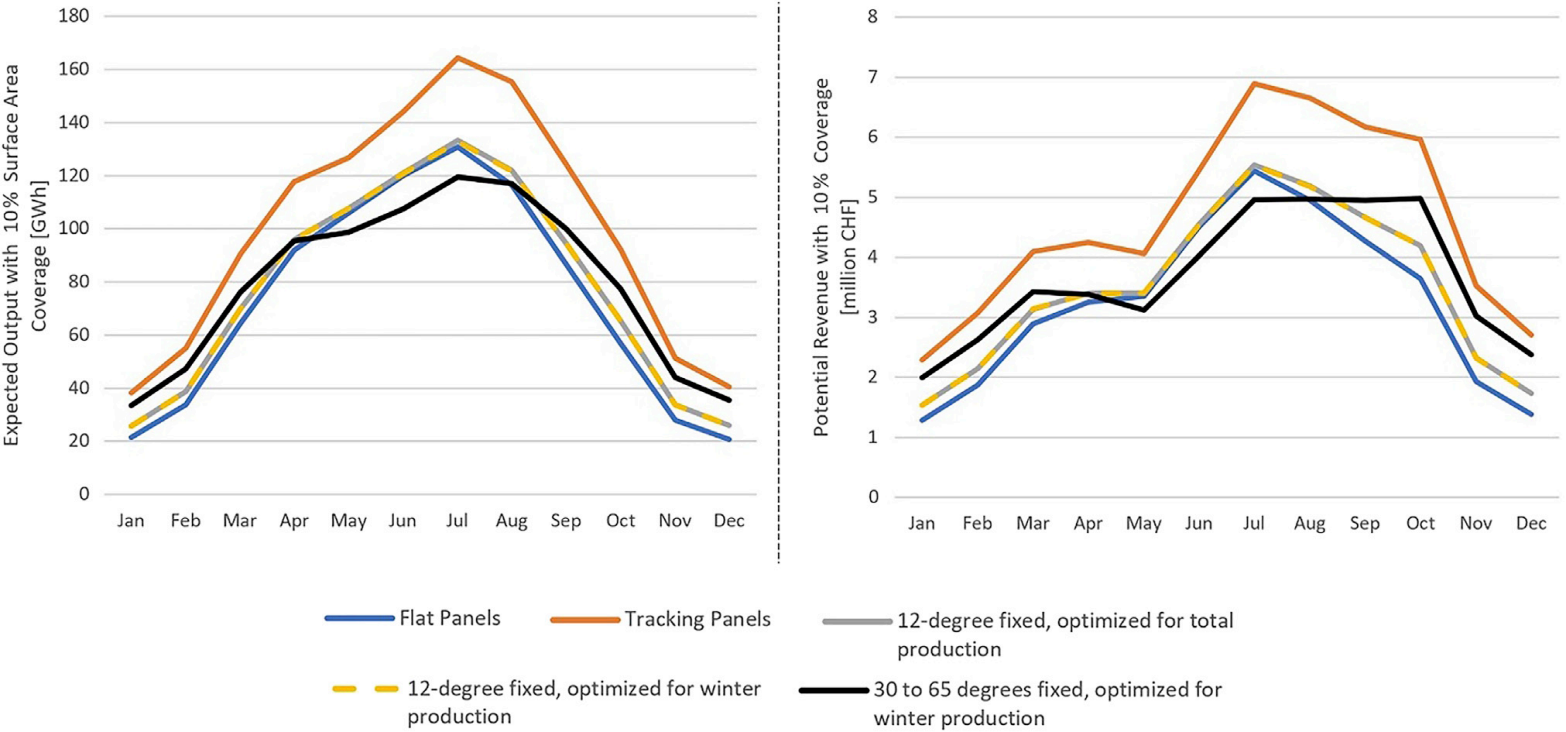
Results for multiple design configurations assuming panel surface area equals 10% of the respective water body’s surface area and 15% efficiency Left: total expected floating solar output. Right: potential total revenue profiles assuming power is sold at the time of generation on the Swiss wholesale market.

Conservative aggregate expected generation profiles over our sample of water bodies indicate that the amount of solar energy radiating on Swiss high-altitude lakes is substantial, with a total amounting to the equivalent of 86.7% of Swiss national electricity consumption for our sample and an annual average of 1.7 MWh per square meter and over 700 GWh per water body per year. The expected annual output with 10% surface area coverage for each investigated system configuration is presented in Figure 7. Our results rate annual tracking output at roughly 1.4 times higher than flat panel output for floating solar in the Swiss Alps. Our azimuth optimizations of fixed panels at a tilt of 12° for total and winter output yielded very similar results, with over a quarter of the sites in our sample showing no azimuth deviation between seasonal optima. As a result, the annual output with panels fixed at 12° is roughly 1.06 times flat output for both cases. Yearly production for the total optimization is merely 0.06% higher than the output when optimized for winter, suggesting that fixed panels should always be optimized for winter production given the higher economic value of winter electricity in Switzerland. Finally, fixed panels optimized for winter output with a tilt between 35 and 60° can produce 1.09 times flat production, with an average tilt of 45.7°. Total generation profiles under each investigated design configuration are presented in Figure 8.

With 100% surface area coverage, systems at 15% efficiency would substitute up to half of Switzerland’s nuclear electricity production in 2018, accounting for between 13 and 18% of Swiss electricity generation. A more feasible 10% surface area coverage would imply that high-altitude floating solar technology would be responsible for between 1.3 and 1.8% of Swiss electricity production. The production spread represents the difference in output between flat panels and tracking systems. Across our sample, the corresponding marginal contribution of each percentage point of site surface area stands at 0.13%–0.18% of Swiss electricity production. With more efficient PV panels in the future, systems will account for greater shares of Swiss generation.

Ranking prospective sites by total expected output reveals that available surface area is the primary factor when determining the technical potential of floating solar power across our sample. *Lac d’Emosson* and *Lac de Salanfe* are identified as the most interesting prospects, as they are the only two sites among the top 10 for both total output and output per square meter. At a 15% efficiency, the total expected annual output with 10% coverage stands at 62.9 GWh for *Lac d’Emosson* and 35.7 GWh for *Lac de Salanfe*, whereas 196 and 199 kWh can be harnessed every year per square meter for the two sites, respectively. These values are significantly higher than the averages across all sites considered in this study, which lie at 10.7 GWh of total output per year with 10% coverage and 178 kWh annually per square meter for flat panels.

Whereas tracking systems dominate all other configurations, fixed panels optimized for winter output with tilts between 30 and 65° can harness an average of 87% of tracking production from November through February. Compared with flat panels, these high tilt angles allow output to be shifted from summer months to the winter season while simultaneously increasing total annual production. Tracking systems and high tilts are also favorable at high altitude as they substantially reduce the amount of snow that can accumulate on the surface, a factor that limits productivity.

Despite the increase in winter generation (November-April) with higher tilts, high-altitude floating solar sites produce most of their power in summer. A maximum of 35% of total output can be produced during winter months with tilts between 30 and 65°, compared with 30% for flat panels. Though there may be operational challenges, combining floating photovoltaics with hydropower through hybridization could save some water during the winter months because the electricity from PV could be used instead of running hydro turbines.

Higher variances in historic output are observed as panel tilt increases, with tracking panels exhibiting significantly higher normalized variance than any other configuration. In addition, lower variances are observed in winter for all cases besides fixed panels between 30 and 65°. As a result, the lowest uncertainty in high-altitude floating solar production is achieved with flat panels, where the annual lower bound for 30-min slots stands at 18% (with 95% confidence). In contrast, the highest uncertainty in output is realized with tracking panels, with a corresponding annual lower bound of 6%.

### Addressing the temporal supply/demand mismatch

Assuming 100% surface area coverage within selected water bodies and 15% efficiency, our sample of 82 sites could alleviate up to a third of the temporal discrepancy between electricity production and consumption in Switzerland. A larger portion of the mismatch can be addressed as panel tilt increases. These results confirm the potential for high-altitude solar arrays to relieve pressure on Switzerland’s electricity market in winter. Moreover, we find that high tilts are not explicitly needed to significantly address the temporal supply/demand discrepancy. Flat panels on our sample of water bodies can account for 85% of the mismatch offset achievable with fixed panels between 30 and 65°. Marginally, covering an additional 1% of each water body’s surface area has the potential to decrease the temporal deficit by roughly 0.4%.

### Substantial CO_2_ offset potential

If 15%-efficient floating solar panels would cover the entire 50.1 square kilometers of our sample, the resulting annual reduction in CO_2_-equivalent emissions would be roughly equivalent to two-thirds of total European emissions from coal power in 2016. Once again, tracking panels dominate, potentially reducing annual CO_2_-equivalent emissions by over 1 gigaton. For comparison, flat panels in this case would decrease emissions by roughly 717 megatons per year. As a reference, between 7.2 and 10.3 megatons of CO_2_ could be offset every year for each percentage of water body coverage. However, it should be noted that these results do not take the full life cycle of floating solar technology into account. Instead, these figures represent annual CO_2_-equivalent offsets as a result of substituting clean electricity for non-zero emissions sources, assuming the floating solar arrays have already been built.

### Economic viability of high-altitude floating solar power

Despite substantial revenue potential on the day-ahead market, high-altitude floating solar power is currently not economically viable without subsidies or conversion efficiencies substantially higher than 15% (assuming power is sold at the time of generation). Although tracking panels and designs with tilts between 30 and 65° boast significantly higher energy yields than flat arrays and panels fixed at 12°, these systems would still be unprofitable on the free market if they could be built at the same costs as 12° arrays. A 50–60% reduction in the capital costs reported in (World Bank Group, 2019) is required for the economic viability of flat panels across our sample. However, these results outline a path toward reducing grid connection costs and increasing competitiveness by taking advantage of the existing grid infrastructure provided by associated hydropower plants.

Following the trend in total production, increased yearly revenues can be attained with higher panel tilts, with an annual total potential revenue ranging between CHF 388 million for flat panels and CHF 551 million for tracking systems, assuming 100% surface area coverage and 15% efficiency (1 CHF ∼1 USD).

Total capital costs for floating solar installations with 10% coverage under flat and 12° tilts roughly correspond to half the cost of installing a new coal power plant (assuming a cost of approximately CHF two billion for a GW-scale coal plant). For reference, this range is equivalent to between CHF 107 million and CHF 124 million for each percentage of surface area coverage. Since our cost model is linear with respect to the surface area, the greatest economic viability is achieved by selecting sites with the highest energy output per square meter.

Ranking locations by energy efficiency (Wh/m^2^) is equivalent to ranking sites via our LCOE results. *Lac d’Emosson* and *Lac de Salanfe* are identified once again as top sites, both technically and economically. As a key insight, our results indicate a general tradeoff in Switzerland between economic viability and technical potential (with the notable exceptions of *Lac d’Emosson* and *Lac de Salanfe*). This tradeoff stems from relatively small surface areas for most sites with high economic viability rankings, resulting in lower technical potential.

At 15% efficiency, LCOE for flat panels range from 0.74 to 3.8 CHF cents per kWh, representing lifetime costs assuming a discount rate between 7 and 10%. Panels fixed at 12° are relatively more expensive, with LCOE values roughly 9% higher than those calculated for flat panels. This implies that flat arrays are the most economically viable design case for current systems, assuming snow cover has been dealt with (it should be noted that panels with higher tilts may be most viable if the accumulation of snow is considered, given that high tilts would allow for snow to slide off the panels (Kahl et al., 2019). Assuming the same costs as 12° designs, tilting panels between 30 and 65° results in a 6% increase in LCOE compared with flat arrays. Of the configuration cases explored in this study, only tracking panels have lower average LCOE values than flat systems, lying 16% below the levelized cost for horizontal arrays if they can be built at the same costs as 12° panels.

### Cost targets for profitable ventures

Table 2 presents cost targets needed for high-altitude floating solar arrays to be lucrative when power is sold at the time of generation. As a baseline, if capital costs reach between 0.41 USD/Wp and 0.51 USD/Wp, flat panels would be economically viable without subsidies across our sample. For tracking systems, cost targets range between 0.58 USD/Wp and 0.71 USD/Wp for economic viability. Panels with a fixed tilt of 12° would be profitable if costs are below 0.54 USD/Wp, whereas tilts of 30–65° require a cost target of 0.56 USD/Wp. Overall, current capital costs would have to decrease by roughly 50–60% for high-altitude floating solar technology to be profitable in Switzerland under our assumptions.

**Table 2:** Baseline cost targets to achieve various levels of economic viability with flat panels

Cost target (USD/Wp)	Implication
0.51	25 out of 82 sites would be economically viable
0.47	Sample as a whole would be economically viable (sum of NPV > 0)
0.41	All 82 sites would be economically viable

Values assume no government subsidies and a discount rate of 7%.

### Global potential for high-altitude floating solar power

**Figure 9:**
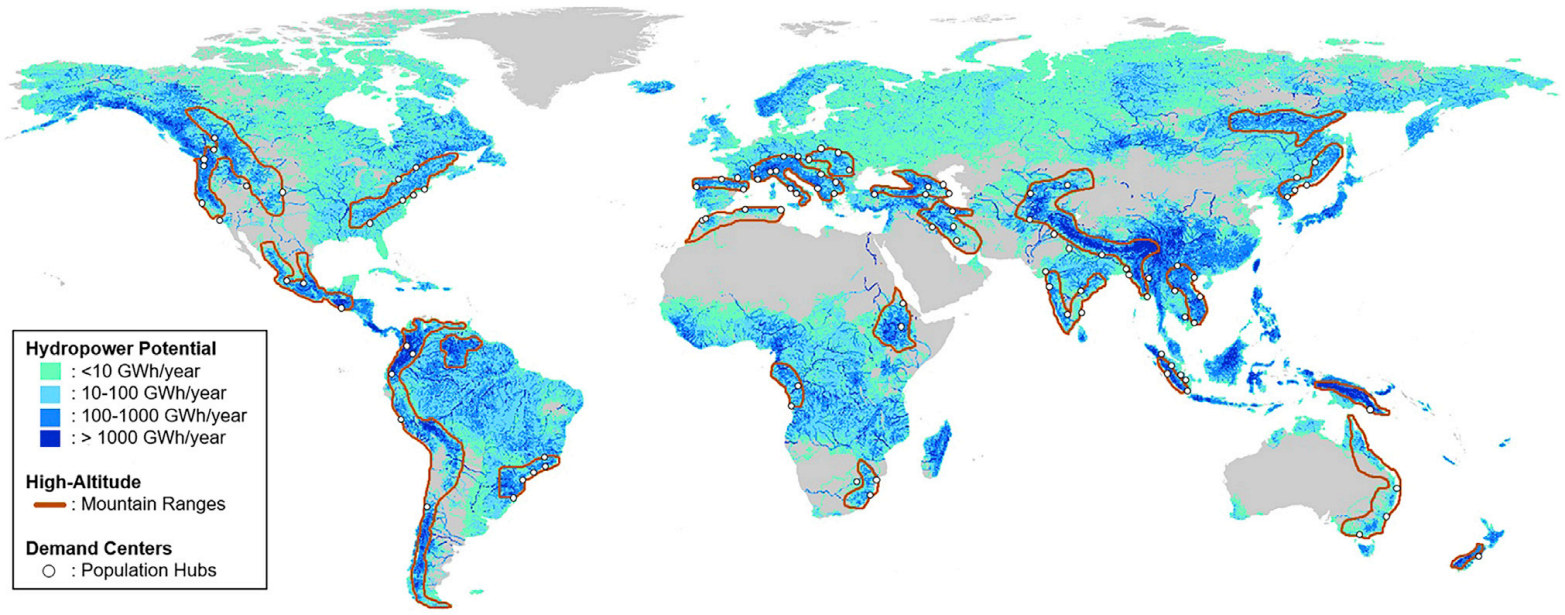
Global perspective for the potential of high-altitude floating solar applications Overlays of global hydropower potential, key/large mountain ranges, and electricity demand centers (population hubs) illustrate areas of interest. This is within the TWh-scale range of global potential for combined PV-hydro systems, without considering the altitude (Lee et al., 2020). Details on the method and data sets can be found in the SI Appendix (Figures S1, S2, and S3).

Figure 9 illustrates hydropower resources, mountain ranges, and electricity demand centers on a global scale. Areas of interest for high-altitude floating solar applications can be found on almost every continent, including many locations with land constraints where the technology could provide greater electricity generation potential than rooftops. As a result, a significant number of locations across the world with existing hydropower dams could benefit from high-altitude floating solar while hedging against lost revenues from seasonal hydropower fluctuations. The remarkable results in Switzerland’s case indicate that these regions should consider high-altitude floating solar power while developing their energy strategies. By demonstrating the suitability of high-altitude floating arrays in the Swiss Alps, the results we present here should serve as a guide for further research on mitigating climate and energy risk through the use of high-altitude floating solar power. Previous research demonstrates that in the UK, when PV is sited at altitudes greater than 6 km, it is possible to produce four times the energy produced by ground-based PV (Aglietti et al., 2009). The map highlights the possibility for further application in hydropower reservoirs and consideration that high-altitude sites in general result in greater electricity generation potential.

## Discussion

With the ability to provide large amounts of power and significantly reduce CO_2_ emissions, emerging floating solar technology could provide land-sparing, low-carbon electricity in high-altitude mountainous regions globally. Our technical results provide compelling motivation for the development of suitable alpine floating solar installations in Switzerland, particularly if existing storage and grid connections are exploited through hybrid floating solar/hydro systems. In addition, the environmental benefits of modular and reversible systems along with significant CO_2_ offsets make a strong case for pursuing this technology under the vision of a sustainable future. The floating solar industry remains in infancy and further research is required to achieve the significant reduction in capital costs or increase in the efficiency needed for economic viability without subsidies or storage. A concerted decarbonization research agenda could utilize these high-performance solar zones to understand integration costs with existing global grid infrastructure. This study offers individual generation profiles and cost criteria for successful projects on 82 suitable water bodies, thereby providing the foundations for the next steps in exploiting high-altitude floating solar technology.

Whereas subsidies for solar power plants are given in many countries, costs may significantly decrease, or power could be sold at higher prices to be economically viable without subsidies – for example, through storage arbitrage as a “baseload” plant. Our model of capital costs includes expenses related to building utility-scale grid connections for each prospective site. Sharing this infrastructure with associated hydropower plants may result in significantly lower construction costs, adding to the expected decrease in costs as the floating solar industry matures. Integrations with existing hydro utilities may also present opportunities for O&M synergies, with the benefits of existing on-site personnel and road access. Additionally, any increases in efficiency would have a positive effect on cost targets. The assumed 15% efficiency may be considerably lower than what is achievable given the low operating temperatures, the natural cooling effect of water, and recent advances in photovoltaic energy conversion technology. Combining high-altitude floating solar with storage technology would also increase site profitability by enabling the sale of generated power at higher prices. This may be achieved through integration with associated hydro pumped-storage facilities. As for the effects of icing, commercial floating solar panels which can withstand icing are already available on the market (Ciel and Terre International, 2019). Industry leader, Ciel & Terre, notes that commercial floating solar withstands icing conditions and can act as a hedge when ice causes issues for hydropower generation (floating solar can continue to generate electricity unless it is completely blocked from light radiation). Whereas the hydropower sites in our sample are currently operational during winter, this could be a key hedging opportunity for hydropower systems operating at very low temperatures.

### Conclusions

The prospect of integrating floating solar panels with hydropower plants is especially relevant as climate change has created uncertainty in future water resources for hydro utilities (Beniston, 2012; Schmitt et al., 2019). Hybrid solar/hydro systems can help stabilize production and mitigate climate risks, with complementary use cases in peaking plants, load balancing, energy arbitrage, and ancillary grid services. Furthermore, floating solar output could be used to compensate for times when water storage levels are low, providing valuable relief for hydro operators. This would result in less reliance on imports during the filling season and increased savings of hydro capacity for the winter. In addition, reduced evaporation rates on hydro reservoirs with floating solar implies further valuable water savings. Hybrid integration of floating solar with hydropower is still at an early stage (World Bank Group, 2019).

Incentives are strong for Swiss hydro managers to pursue floating solar systems using their existing substation infrastructure given the potential benefits and strategic importance of integration, especially as the floating solar industry matures. As an example, *Statkraft* (a large Norwegian hydro producer) is optimistic about complementing hydro production with floating solar and is currently pursuing pilot integrations in Albania and other operations are occurring across Thailand (CleanTechnica, 2019; Clemons et al., 2021). In addition, the increased revenue potential from floating solar combined with hydro storage (enabling power sales at higher prices) also provides further prospects for viable business cases and room for innovation through research, demonstration, and deployment (Kittner et al., 2017).

Besides cost barriers and engineering, the implementation of high-altitude floating solar faces several challenges. Public opinion may be against such projects, especially since many of the water bodies in our sample are popular tourist destinations. Previous research on the risks of developing photovoltaic projects in the Swiss alps has found that project acceptance relies heavily on contributions to the local economy, with transparent and regular information flows between stakeholders as a key driver of project approval (Díaz and Van Vliet, 2018) – the study also found that the high complexity of administrative processes related to developing new renewable projects pose a significant implementation risk. Given the existing local relationships and permits held by hydropower facilities, the path of least resistance for implementing high-altitude floating solar is likely to be through associated hydro operators. At higher levels of PV integration, grid operators may need to change traditional habits to ensure system stability – despite adequate transmission interconnections and co-location of pumped hydro storage resources, there may need to be new grid management strategies to achieve cost-effective integration of new floating solar resources.

Finally, from a global perspective, high-altitude sites may be difficult to access in some locations. Remote areas that do not have existing transmission system infrastructure or an existing hydropower reservoir would be difficult to access. Our map in Figure 9 identifies areas that would be easier to approach. However, the maintenance for high-altitude panels to reduce snow or dust cover could be costlier than for a ground-mounted utility-scale solar installation. Overall, our results suggest that high-altitude floating solar technology should be on the global radar for alternative utility-scale solar electricity technologies. The prospect of utility-scale production and homogeneous spaces presents the technology as a solid option for large-scale expansions in mountainous regions. Specifically, Swiss hydropower managers have much to gain by incorporating floating solar systems. Pilot projects on *Lac d’Emosson* and *Lac de Salanfe*, the top 2sites identified in our analysis, would be an appropriate starting point for the roll-out of high-altitude floating solar arrays in Switzerland.

The technical potential and economic benefits of high-altitude floating solar technology have been demonstrated to be highly promising across high-altitude regions. Key barriers to implementation include substantial capital costs, which are currently still too high for economic viability without subsidies or storage, and engineering challenges in tailoring the technology to alpine water bodies. However, Switzerland and many other regions are well poised to exploit high-altitude floating solar power in the near future if investments are made in research and development of utility-scale projects. Costs are expected to drop and the added value for the Swiss hydropower sector presents high-altitude floating solar as a strategic opportunity to reduce risk and reliance on imports – serving as an example for the rest of the world.

### Limitations of the study

For the proliferation of high-altitude floating solar power, further research is needed to determine the most suitable design configuration. Although current products are capable of withstanding heavy winds and snowfall (Ciel and Terre International, 2019), snow-covered panels result in decreased efficiency (Awad et al., 2018). This implies lower production during winter months, precisely when it is desirable to maximize output to alleviate the temporal supply/demand mismatch. Current research is exploring the use of hydrophobic and ice-phobic coatings to avoid snow cover, whereas the ability of high-tilts to significantly reduce the accumulation of snow on solar panels has been demonstrated (Andenæs et al., 2018). The use of bifacial panels is a particularly interesting topic to explore, as such systems could exploit reflections from the water surface to boost generation while using high tilts to increase winter output. This study concludes that flat panels are currently the most economically viable option, assuming the accumulation of snow is dealt with through maintenance. However, at the right costs, higher tilts and tracking systems would be able to make a larger impact and produce more valuable electricity in winter. On an annual basis, tracking panels produce roughly 40% more power than flat panels, whereas fixed-tilts increase generation by 6 and 9% for 12° panels and tilts between 30 and 65°, respectively. The increased investment needed for these systems may be justified by gains in production, especially considering the importance of adding winter capacity. Technically speaking, tracking panels and tilts between 30 and 65° are the most promising configurations we investigated. However, the application of such systems in harsh conditions at high altitude requires further research.

## STAR★Methods

### Key resources table

**Table:** 

REAGENT or RESOURCE	SOURCE	IDENTIFIER
**Deposited data**		

Swiss water body data (coordinates, altitude, surface area, dams under federal supervision)	Swiss Federal Office of Topography (swisstopo)	https://map.geo.admin.ch/
Swiss hydropower facility data (coordinates, associated water bodies, plant type)	Swiss Federal Office of Energy	https://www.bfe.admin.ch/bfe/en/home/versorgung/statistik-und-geodaten/energiestatistiken/teilstatistiken.exturl.html/aHR0cHM6Ly9wdWJkYi5iZmUuYWRtaW4uY2gvZGUvcHVibGljYX/Rpb24vZG93bmxvYWQvOTY5MA==.html
Surface incoming shortwave irradiance	EUMETSAT Satellite Application Facility on Climate Monitoring	https://doi.org/10.5676/EUM_SAF_CM/SARAH/V002_01
Surface incoming direct irradiance	EUMETSAT Satellite Application Facility on Climate Monitoring	https://doi.org/10.5676/EUM_SAF_CM/SARAH/V002_01
Surface albedo	EUMETSAT Satellite Application Facility on Climate Monitoring	https://doi.org/10.5676/EUM_SAF_CM/CLARA_AVHRR/V002
Swiss electricity production and consumption data	Swissgrid	https://www.swissgrid.ch/dam/dataimport/energy-statistic/EnergieUebersichtCH-2018.xls
Swiss day-ahead electricity prices	ENTSO-E Transparency Platform	https://transparency.entsoe.eu

**Software and algorithms**		

HASPR Python Suite	Purposefully built for this study (N. Eyring)	https://github.com/bonesbb/HASPR
Python implementation of Solar Position Algorithm	Pysolar Development Team	http://docs.pysolar.org/en/latest

### Resource availability

#### Lead contact

Further information can be directed to Dr. Noah Kittner (kittner@unc.edu).

#### Materials availability

This study did not generate new physical materials.

### Method details

#### Note on execution time and hardware

Using brute-force to optimize fixed-tilt positions in Cases 3, 4, and 5 requires us to compute a very large number of historic generation profiles. Our implementation of all models described herein can run to scale with as little as 1 GB of RAM. However, the bottleneck lies in the CPU. Typical execution time for a model thread can be up to 15 minutes per generation profile per year on a modern processor. To speed things up, we executed in parallel batches on the Euler cluster at ETH Zürich. Respecting the usage limits of Euler for non-shareholders, acceleration was achieved by slicing our models into 30 standard batch jobs at a time, each calculating 20 generation profiles and running in 4-hour parallel slots. This framework implies a maximum capacity of 600 yearly 30-minute resolution generation profiles computed every 4 hours. A total of 1604 batch jobs were executed on Euler for this study, amounting to approximately 250 days of processor time.

#### Note on missing data values

Our meteorological satellite data was obtained through highly-sensitive geostationary instruments with a non-zero probability of downtime. This results in multiple Not A Number (NAN) data points. The number of NAN values in the retrieved data differs from year to year and depends on the coordinates of the site in question. Our SIS and SID values are calibrated and validated by CMSAF. However, NANs persist in the data sets. To take the inconsistency of the satellite data into account, HASPR includes the total number of NANs in each generation profile’s output and conservatively sets all NANs to zero.

#### Analyzing the global potential of high-altitude floating solar

We conducted an overlay analysis to discover locations outside of Switzerland which could benefit from the introduction of high-altitude floating solar power. This analysis was carried out in three steps:

Step 1: Global map of hydropower potential (supplemental information) – based on data from (Hoes et al., 2017). Hydropower potential was selected as a starting point to indicate the locations of potential sites for floating solar applications.
Step 2: Global map of mountain ranges (supplemental information) – based on data from (Elsen and Tingley, 2015). The perimeters of key/large mountain ranges allow us to outline a baseline for global high-altitude hydropower resources.
Step 3: Global map of population density (supplemental information) – based on data from (Schiavina et al., 2020). High population density implies substantial electricity demand. For our analysis, we pinpoint population hubs which are found within or in the vicinity of high-altitude hydropower resources.

#### HASPR python suite – Readme

HASPR grants users the ability to model the output of solar arrays given high-resolution meteorological data.

High-Altitude Solar Power Research (HASPR) - Case Study of High-Altitude Floating Solar in Switzerland.

##### Developer: Nicholas eyring (neyring)

HASPR’s scripts are segmented in two parts. The first part calculates generation profiles for sets of coordinates at a temporal resolution of 30 minutes and is the computational bottleneck. The second part consists of analysis scripts which are computationally insignificant in comparison with our POA model. This being the case, HASPR’s structure is designed to execute the first part on a high-performance computer and to execute the second part on a typical workstation. Currently, HASPR consists of a collection of scripts which can be challenging to configure for less advanced users. For help with getting started in HASPR, get in touch at eyring.nick@gmail.com.

#### Core scripts

*datascrape*.*py*: Script to extract light-weight data sets and merge files from large NetCDF4 directories.
*haspr*.*py*: Background script/library containing classes, functions, and global variables.

#### Generation scripts

*batch_check*.*py*: Checks if batches have successfully run. Outputs incomplete batch list.
*batch_submission_bf*.py: Script for setting up brute force batch jobs for fixed-tilt optimizations on Euler.
*batch_submission_opt*.py: Script for setting up batch jobs for fixed-tilt calculations on Euler.
*main_euler_fixed*.*py*: Main script for Euler fixed-tilt batches. Sets parameters, runs models, and dumps data.
*main_euler_flat*.*py*: Main script for Euler flat batches. Sets parameters, runs models, and dumps data.
*main_euler_tracking*.*py*: Main script for Euler tracking batches. Sets parameters, runs models, and dumps data.
*optimization_results*.*py*: Determines optimum fixed-tilt positions given directories of brute force output.
*organize_batch_output*.*py*: Copies files from batch output to corresponding historic profile directories.

#### Processing scripts

*global_remove_leap*.*py*: Removes leap days for a global panel configuration case.
*lower_resolution*.*py*: Converts data series to hourly, daily, or monthly resolution.
*remove_leap_days*.*py*: Script to remove leap days from a directory of generation profiles.

#### Analysis scripts

*average_aggregate_revenue*.*py*: Outputs average bid and potential revenue given a directory of aggregate revenue profiles.
*average_individual_revenue*.*py*: Script which averages individual revenue profiles from historic data.
*co2_offset*.*py*: Calculates CO2-equivalent offset given generation profiles.
*expected_output_analysis*.*py*: Computes aggregate lower bounds and historic variance given a directory of individual expected output.
*expected_site_output*.*py*: Script to calculate expected output for one site given a directory of historic profiles.
*global_expected_site_output*.*py*: Script to calculate expected output for all sites under a design configuration.
*lifetime_costs*.*py*: Calculates costs given a CSV file of sites, panel surface area, and tilt angles.
*lifetime_revenue*.*py*: Calculates yearly and cumulative revenue for system lifetime given a directory of revenue profiles.
*NPV_LCOE*.*py*: Computes the NPV and LCOE given lifetime costs/revenues and expected generation profiles.
*revenue*.*py*: Outputs revenue profiles for all generation profiles in a given directory.
*sum_individual*.*py*: Script to calculate annual sums given generation profiles.
*supply_demand_mismatch*.*py*: Computes the potential alleviation of supply/demand mismatches given generation profiles.
*total_expected_output*.*py*: Script to calculate generation profiles in Wh from a directory of profiles in Wh per square meter.
*total_generation_profile*.*py*: Script to calculate aggregate generation profiles given surface areas.

#### Adding a model to HASPR

Make the following adjustments to *haspr*.*py*:

• Add the model to the “initialize” function, add the required datasets here as well
• Add path variables and *Dataset* class adjustments if new dataset was added
• Write the model’s function (adding results to the model’s “results” array)
• Add code to call the new function if the model’s name matches (in the *Model* class’ execute function)

#### Feeding a list of coordinates to HASPR

HASPR’s solar research models generate results based on a set of coordinates provided by the user. This list of sites of interest needs to be prepared in the form of a .csv file with two columns:

Column 1 = Site ID (integer)
Column 2 = WGS 84 coordinates (string)

HASPR’s *set_coordinates(path)* function takes the file path of the list of coordinates in .csv form as its only argument. This function converts the WGS 84 coordinate string into decimal latitudes and longitudes before setting *haspr*.*py*’s global variable coordinates – an iterable list of each site’s latitude, longitude, and integer ID. HASPR’s generation profile models automatically calculate the output for each site in the coordinates list.

#### Creating batches to accelerate HASPR models

We grant HASPR users the ability to segment their models’ parameter sweeps into multiple batches by setting the *sweep_range* field in the *haspr*.*Model* class. HASPR’s *get_sweep_batches(full_sweep*, *batch_length)* function returns an array of all sweep batches given the full sweep array and batch length as input. A researcher can then set-up multiple *Model* instances with the desired sweep ranges to run in parallel - exploiting HPC technologies to minimize runtime.

#### Euler script documentation

The HASPR library contains three scripts for running batches on the Euler supercomputer: *main_euler_flat*.*py* for obtaining flat generation profiles (Case 1 – maximum 450 sites per batch), *main_euler_tracking*.*py* for obtaining full-tracking generation profiles (Case 2 – maximum 20 sites per batch), and *main_euler_fixed*.*py* for calculating fixed-tilt generation profiles (Cases 3 and 4 – maximum 20 sites per batch). The user defines individual jobs using the command line arguments described below:

- Flat Generation Profiles – *main_euler_flat*.*py*:

##### Command line use

$ bsub python ./main_euler_flat.py [coords] [outputDir] [SISpath]

##### Description of arguments

*coords*: Path to .csv coordinates file containing max 450 sites
*outputDir*: Path to desired output directory (e.g. …/out; needs to be created and empty beforehand)
*SISpath*: Path to SIS dataset (e.g. 2015 SIS data)

##### Example use

$ bsub python ./main_euler_flat.py coords/coords1_to_33.csv output/B6 datasets/2013/00_2013_SIS_merged.nc

- Full-Tracking Generation Profiles – *main_euler_tracking*.*py*:

##### Command line use

$ bsub python ./main_euler_tracking.py [coords] [outputDir] [SISpath] [SIDpath]

##### Description of arguments

*coords*: Path to .csv coordinates file containing max 20 sites
*outputDir*: Path to desired output directory (e.g. …/out; needs to be created and empty beforehand)
*SISpath*: Path to SIS dataset (e.g. 2015 SIS data)
*SIDpath*: Path to SID dataset (e.g. 2015 SID data)

##### Example use

$ bsub python ./main_euler_tracking.py coords/coords21_to_33.csv output/B14 datasets/2009/00_2009_SIS_merged.nc datasets/2009/01_2009_SID_merged.nc

- Fixed-Tilt Generation Profiles – *main_euler_fixed*.*py*:

##### Command line use

$ bsub python ./main_euler_fixed.py [coords] [outputDir] [SISpath] [SIDpath] [optType] [sweepIndex]

##### Description of arguments

*coords*: Path to .csv coordinates file containing 1 site (see Figure xx for format)
*outputDir*: Path to desired output directory (e.g. …/out; needs to be created and empty beforehand)
*SISpath*: Path to SIS dataset (e.g. 2017 SIS data)
*SIDpath*: Path to SID dataset (e.g. 2017 SID data)
*optType*: Optimization type (1 = azimuth sweep at a fixed 12-degree tilt, 2 = full sweep between 30 and 65 degree tilts)
*sweepIndex*: Index for sweep range (first index = 0; each index represents 20 profiles)

##### Example use

$ bsub python ./main_euler_fixed.py coords/coords1.csv output/B31 datasets/2017/00_2017_SIS_merged.nc datasets/2017/01_2017_SID_merged.nc 1 0

Note: When running main_euler_fixed.py to calculate historic profiles after finding the optimum position, simply omit the *optType* and *sweepIndex* arguments and add the optimum azimuth and tilt in columns 3 and 4, respectively, of the .csv coordinates file. Instead of sweeping through positions in this case, the user can input 20 sites at once via the *coords* argument to define a batch.

Note: The path to the SAL dataset is hardcoded into the three Euler scripts since the entire dataset (spanning the years 2006-2015) is small enough to handle with one file.

Note: For memory management reasons, batches only incorporate one year of data (i.e. a batch will be defined for multiple sites over the same year instead of multiple years for one site).

## Data Availability

A summary of the data sets used in our analysis can be found in the Key resources table and supplemental information. All data sets besides values for Switzerland’s electrical grid carbon intensity can be retrieved with no restrictions via the URLs listed. The grid carbon intensity data we used can be acquired by contacting the authors/publisher of (Chevrier et al., 2019) while the collection of Python scripts, individual profiles and supporting documents used for our analysis can be found at https://github.com/bonesbb/HASPR. To establish our sample of potential floating solar sites, Swiss water body data was sourced directly from the *Swiss Federal Office of Topography swisstopo* (swisstopo) via their interactive map of official survey and geological data sets (Swiss Federal Office of Topography Swisstopo, 2019) while the associated Swiss hydropower plant data was retrieved from the yearly hydro statistics report published by the *Swiss Federal Office of Energy* (Swiss Federal Office of Energy, 2019). To calculate historic generation profiles, solar position was computed via *Pysolar* – a python implementation of the Solar Position Algorithm (Pysolar Development Team, 2019) – with the rest of our high-resolution climate data being provided by the *EUMETSAT Satellite Application Facility on Climate Monitoring* (CMSAF) (Pfeifroth et al., 2019; Karlsson et al., 2019). To analyze the Swiss electricity supply/demand mismatch, high-resolution data on total Swiss electricity consumption and production was retrieved from *Swissgrid*, the Swiss transmission system operator (Swissgrid, 2019). For our revenue analysis, Swiss electricity price data was sourced from the *European Network of Transmission System Operators for Electricity* (ENTSO-E) (ENTSO-E Transparency Platform, 2019). Finally, Swiss grid carbon intensity data for our CO2-offset analysis was retrieved from an *ETH Zürich* study distributed by the *Swiss Federal Laboratories for Materials Science and Technology* (EMPA) (Chevrier et al., 2019).

## Footnotes

Supplemental information can be found online at https://doi.org/10.1016/j.isci.2022.104394.

## References

[bib1] Abrell J. (2019). The Swiss Wholesale Electricity Market. https://ethz.ch/content/dam/ethz/special-interest/mtec/cer-eth/economics-energy-economics-dam/documents/people/jabrell/.

[bib2] Aglietti G.S., Redi S., Tatnall A.R., Markvart T. (2009). Harnessing high-altitude solar power. IEEE Trans. Energy Convers..

[bib3] Andenæs E., Jelle B.P., Ramlo K., Kolås T., Selj J., Foss S.E. (2018). The influence of snow and ice coverage on the energy generation from photovoltaic solar cells. Solar Energy.

[bib4] Antonanzas J., Osorio N., Escobar R., Urraca R., Martinez-de-Pison F.J., Antonanzas-Torres F. (2016). Review of photovoltaic power forecasting. Solar Energy.

[bib5] Awad H., Gül M., Salim K.M.E., Yu H. (2018). Predicting the energy production by solar photovoltaic systems in cold-climate regions. Int. J. Sustain. Energy.

[bib6] Bartlett S., Dujardin J., Kahl A., Kruyt B., Manso P., Lehning M. (2018). Charting the course: a possible route to a fully renewable Swiss power system. Energy.

[bib7] Beniston M. (2012). Impacts of climatic change on water and associated economic activities in the Swiss Alps. J. Hydrol..

[bib8] Blumthaler M., Blumthaler M. (2012). Plants in Alpine Regions.

[bib9] Campana P.E., Wästhage L., Nookuea W., Tan Y., Yan J. (2019). Optimization and assessment of floating and floating-tracking PV systems integrated in on- and off-grid hybrid energy systems. Solar Energy.

[bib10] Cazzaniga R., Rosa-Clot M., Rosa-Clot P., Tina G.M. (2019). Integration of PV floating with hydroelectric power plants. Heliyon.

[bib11] Chevrier A., Smith R., Bollinger A. (2019). Calculation of a dynamic carbon factor for the Swiss electricity grid. https://hues.empa.ch/images/Alice_Chevrier_Semester_Project.pdf.

[bib12] Chitturi S.R.P., Sharma E., Elmenreich W. (2018). Efficiency of photovoltaic systems in mountainous areas. IEEE Int. Energy Conf..

[bib13] Ciel and Terre International (2019). Hydrelio: The Patented Floating PV System. https://www.ciel-et-terre.net/hydrelio-floating-solar-technology/hydrelio-products/.

[bib14] CleanTechnica (2019). What’s not to love about floating solar farms. https://cleantechnica.com/2019/05/13/whats-not-to-love-about-floating-solar-farms-cleantechnica-interview/.

[bib15] Clemons S.K.C., Salloum C.R., Herdegen K.G., Kamens R.M., Gheewala S.H. (2021). Life cycle assessment of a floating photovoltaic system and feasibility for application in Thailand. Renew. Energy.

[bib16] Darling S.B., You F., Veselka T., Velosa A. (2011). Assumptions and the levelized cost of energy for photovoltaics. Energy Environ. Sci..

[bib17] Davis S.J., Lewis N.S., Shaner M., Aggarwal S., Arent D., Azevedo I.L., Benson S.M., Bradley T., Brouwer J., Chiang Y.M. (2018). Net-zero emissions energy systems. Science.

[bib18] Díaz P., Van Vliet O. (2018). Drivers and risks for renewable energy developments in mountain regions: a case of a pilot photovoltaic project in the Swiss Alps. Energy Sustain. Soc..

[bib19] Dubey S., Sarvaiya J.N., Seshadri B. (2013). Temperature dependent photovoltaic (PV) efficiency and its effect on PV production in the world – a review. Energy Proced..

[bib20] Dujardin J., Kahl A., Kruyt B., Bartlett S., Lehning M. (2017). Interplay between photovoltaic, wind energy and storage hydropower in a fully renewable Switzerland. Energy.

[bib21] Elsen P.R., Tingley M.W. (2015). Global mountain topography and the fate of montane species under climate change. Nat. Clim. Change.

[bib22] ENTSO-E Transparency Platform (2019). Day-ahead Prices - Switzerland. https://transparency.entsoe.eu.

[bib23] Er Z., Rouabah Z., Kizilkan G., Orken A.T. (2018).

[bib24] Gorjian S.H., Sharon H., Ebadi H., Kant K., Scavo F.B., Tina G.M. (2020). Recent technical advancements, economics and environmental impacts of floating photovoltaic solar energy conversion systems. J. Clean. Prod..

[bib25] Hansen J., Sato M., Hearty P., Ruedy R., Kelley M., Masson-Delmotte V., Russell G., Tselioudis G., Cao J., Rignot E. (2016). Ice melt, sea level rise and superstorms: evidence from paleoclimate data, climate modeling, and modern observations that 2 C global warming could be dangerous. Atmos. Chem. Phys..

[bib26] Hay J.E. (1993). Calculating solar radiation for inclined surfaces: practical approaches. Renew. Energy.

[bib27] Hoes O.A.C., Meijer L.J.J., Van Der Ent R.J., Van De Giesen N.C. (2017). Systematic high-resolution assessment of global hydropower potential. PLoS One.

[bib28] Jinko Solar Team (2021). Tiger Pro 72HC 530-550 Watt Mono-Facial Module. https://www.jinkosolar.com/uploads/5ff587a0/JKM530-550M-72HL4-(V)-F1-EN.pdf.

[bib29] Jurasz J., Dąbek P.B., Kaźmierczak B., Kies A., Wdowikowski M. (2018). Large scale complementary solar and wind energy sources coupled with pumped-storage hydroelectricity for Lower Silesia (Poland). Energy.

[bib30] Jurasz J., Mikulik J., Krzywda M., Ciapała B., Janowski M. (2018). Integrating a wind-and solar powered hybrid to the power system by coupling it with a hydroelectric power station with pumping installation. Energy.

[bib31] Kahl A., Dujardin J., Lehning M. (2019). The bright side of PV production in snow-covered mountains. Proc. Natl. Acad. Sci. U S A.

[bib32] Kamuyu W.C.L., Lim J.R., Won C.S., Ahn H.K. (2018). Prediction model of photovoltaic module temperature for power performance of floating PVs. Energies.

[bib33] Karlsson K.G., Anttila K., Trentmann J., Stengel M., Meirink J.F., Devasthale A., Hanschmann T., Kothe S., Jääskeläinen E., Sedler J. (2019). CLARA-A2: CM SAF Cloud, Albedo and surface radiation dataset from AVHRR Data - Edition 2.

[bib34] Kern J., Harris I. (1975). On the optimum tilt of a solar collector. Solar Energy.

[bib35] Khiareddine A., Ben Salah C., Rekioua D., Mimouni M.F. (2018). Sizing methodology for hybrid photovoltaic/wind/hydrogen/battery integrated to energy management strategy for pumping system. Energy.

[bib36] Kittner N., Castellanos S., Hidalgo-Gonzalez P., Kammen D.M., Kurtz S. (2021). Cross-sector storage and modeling needed for deep decarbonization. Joule.

[bib37] Kittner N., Gheewala S.H., Kammen D.M. (2016). Energy return on investment (EROI) of mini-hydro and solar PV systems designed for a mini-grid. Renew. Energy.

[bib38] Kittner N., Lill F., Kammen D.M. (2017). Energy storage deployment and innovation for the clean energy transition. Nat. Energy.

[bib39] Kougias I., Bodis K., Jager-Waldau A., Monforti-Ferrario F., Szabo S. (2016). Exploiting existing dams for solar PV system installations. Prog. Photovolt..

[bib40] Kougias I., Szabo S., Monforti-Ferrario F., Huld T., Bodis K. (2016). A methodology for optimization of the complementarity between small-hydropower plants and solar PV systems. Renew. Energy.

[bib41] Lave M., Hayes W., Pohl A., Hansen C.W. (2015). Evaluation of global horizontal irradiance to plane-of-array irradiance models at locations across the United States. IEEE J. Photovolt..

[bib42] Lee N., Grunwald U., Rosenlieb E., Mirletz H., Aznar A., Spencer R., Cox S. (2020). Hybrid floating solar photovoltaics-hydropower systems: benefits and global assessment of technical potential. Renew. Energy.

[bib43] Li Y., Gao W., Ruan Y., Ushifusa Y. (2018). The performance investigation of increasing share of photovoltaic generation in the public grid with pump hydro storage dispatch system, a case study in Japan. Energy.

[bib45] Pfeifroth U., Kothe S., Trentmann J., Hollmann R., Fuchs P., Kaiser J., Werscheck M. (2019). Surface radiation data set - heliosat (SARAH) - edition 2.1.

[bib46] Pysolar Development Team (2019). Pysolar Python Library. http://docs.pysolar.org/en/latest/.

[bib47] Quaranta E., Aggidis G., Boes R.M., Comoglio C., De Michele C., Ritesh Patro E., Georgievskaia E., Harby A., Kougias I., Muntean S. (2021). Assessing the energy potential of modernizing the European hydropower fleet. Energy Convers. Manag..

[bib48] Ranjbaran P., Yousefi H., Gharehpetian G.B., Astaraei F.R. (2019). A review on floating photovoltaic (FPV) power generation units. Renew. Sustain. Energy Rev..

[bib49] Rosa-Clot M., Tina G.M. (2020). Floating PV Plants.

[bib50] Bontempo Scavo F., Tina G.M., Gagliano A., Nizetic S. (2021). An assessment study of evaporation rate models on a water basin with floating photovoltaic plants. Int. J. Energy Res..

[bib51] Schiavina M., Freire S., Kytt M. (2020). GHS Population Grid Multitemporal (1975, 1990, 2000, 2015) R2019A. http://data.europa.eu/89h/0c6b9751-a71f-4062-830b-43c9f432370f.

[bib52] Schmitt R.J.P., Kittner N., Kondolf G.M., Kammen D.M. (2019). Deploy diverse renewables to save tropical rivers. Nature.

[bib53] Shan R., Reagan J., Castellanos S., Kurtz S., Kittner N. (2022). Evaluating emerging long-duration energy storage technologies. Renew. Sustain. Energy Rev..

[bib54] Silvério N.M., Barros R.M., Tiago Filho G.L., Redón-Santafé M., Santos I.F.S.D., Valério V.E.D.M. (2018). Use of floating PV plants for coordinated operation with hydropower plants: case study of the hydroelectric plants of the São Francisco River basin. Energy Convers. Manag..

[bib55] Spencer R.S., Macknick J., Aznar A., Warren A., Reese M.O. (2018). Floating photovoltaic systems: assessing the technical potential of photovoltaic systems on man-made water bodies in the continental United States. Environ. Sci. Technol..

[bib56] Sukarso A.P., Kim K.N. (2020). Cooling effect on the floating solar PV: performance and economic analysis on the case of West Java Province in Indonesia. Energies.

[bib57] Swiss Federal Office of Energy (2018). Schweizerische Elektrizitätsstatistik. https://www.bfe.admin.ch/bfe/en/home/supply/statistics-and-geodata/energy-statistics.html.

[bib58] Swissgrid (2019). Energy Statistic Switzerland. https://www.swissgrid.ch/dam/dataimport/energy-statistic/EnergieUebersichtCH-2018.xls.

[bib59] Swisstopo (2019). Interactive Map of Official Survey and Geological Data Sets. https://map.geo.admin.ch/.

[bib60] U.S. Energy Information Administration (2019). CO2 Emissions from Coal - Europe. https://www.eia.gov/beta/.

[bib61] World Bank Group (2019). Where Sun Meets Water: Floating Solar Market Report - Executive Summary. http://documents.worldbank.org/curated/en/579941540407455831/pdf/Floating-Solar-Market-Report-Executive-Summary.pdf.

[bib62] Xu X., Hu W., Cao D., Huang Q., Chen C., Chen Z. (2020). Optimized sizing of a standalone PV-wind hydropower station with pumped-storage installation hybrid energy system. Renew. Energy.

[bib63] Yang D. (2016). Solar radiation on inclined surfaces: corrections and benchmarks. Solar Energy.

