# Supplementary material for: High-resolution electricity generation model demonstrates suitability of high-altitude floating solar power
Source: iScience. 2022 May 13;25(6):104394. doi: 10.1016/j.isci.2022.104394 (PMC9157236; doi:10.1016/j.isci.2022.104394)
Supplement: Document S1. Figures S1–S3 and Tables S1–S8 [file mmc1.pdf]

iScience, Volume *25*

Supplemental information

High-resolution electricity generation model demonstrates suitability of high-altitude floating solar power

Nicholas Eyring and Noah Kittner

Table S1. Summary of data sets and sources, Related to STAR Methods.

Value	Time Period	Temporal Resolution	Spatial Resolution	Data Source
Water Bodies				
Coordinates	-	-	-	(Swiss Federal Office of Topography swisstopo, 2019)
Altitude	-	-	-	(Swiss Federal Office of Topography swisstopo, 2019)
Surface area	-	-	-	(Swiss Federal Office of Topography swisstopo, 2019)
Dams under Swiss federal supervision	3 July 2018	-	-	(Swiss Federal Office of Topography swisstopo, 2019)
Hydro Installations				
Coordinates	1 January 2018	-	-	(Swiss Federal Office of Energy, 2019)
Associated water bodies	1 January 2018	-	-	(Swiss Federal Office of Energy, 2019)
Plant type	1 January 2018	-	-	(Swiss Federal Office of Energy, 2019)
Meteorological Data				
Surface incoming shortwave irradiance	2008 - 2017	30 minutes	0.05 x 0.05 deg	(Pfeiroth, et al., 2019)
Surface incoming direct irradiance	2008 - 2017	30 minutes	0.05 x 0.05 deg	(Pfeiroth, et al., 2019)
Surface albedo	2006 - 2015	5 days	0.25 x 0.25 deg	(Karlsson et al., 2019)
Solar position (altitude, azimuth)	-	-	-	(Pysolar Development Team, 2019)
Electricity Market				
Total Swiss electricity consumption	2018	15 minutes	-	(Swissgrid, 2019)
Total Swiss electricity production	2018	15 minutes	-	(Swissgrid, 2019)
Swiss day-ahead electricity prices	2015 - 2018	1 hour	-	(ENTSO-E Transparency Platform, 2019)
Grid carbon intensity (CO2-equivalent)	2015	1 hour	-	(Chevrier et al., 2019)

Table S2. Summary of the sample of water bodies used for our analysis, Related to STAR Methods.

Number of Water Bodies	Total Surface Area (km²)	Average Surface Area (km²)	Average Altitude (m)
82	50.1	0.61	1783

Table S3A. Full water body sample and collected attributes, Related to STAR Methods. *: Data for associated hydro installations can be found in (Swiss Federal Office of Energy, 2019).

Site ID	Name	Canton	Altitude (m)	Coordinates (WGS 84)	Surface Area (m²)	Associated Hydro Installations (ZE-Nr.) *
1	Lago Bianco	GR	2234	46°24′18.518″N 10°01′10.443″E	1431937	700200, 700100, 700300, 700400
2	Lac des Dix	VS	2362	46°03′26.019″N 7°23′48.246″E	3999336	505000, 504950, 505100
3	Zmuttbach	VS	1968	46°00′27.630″N 7°42′26.388″E	39741	504600, 504700
4	Grimselsee	BE	1909	46°33′58.305″N 8°18′14.370″E	2687955	200300, 200100, 200400, 200800
5	Oberaarsee	BE	2303	46°32′37.302″N 8°15′32.158″E	1626936	200200, 200100
6	Triebtenseewli	BE	2365	46°33′09.131″N 8°17′52.441″E	96595	200200
7	Totesee	VS	2160	46°33′37.864″N 8°20′24.409″E	193451	200400
8	Räterichsbodensee	BE	1767	46°35′07.786″N 8°19′40.578″E	657746	200500, 201100, 200800, 201300, 200600
9	Gelmersee	BE	1849	46°36′56.434″N 8°19′48.164″E	616993	200400, 200800
10	Lac de Mauvoisin	VS	1969	45°58′46.819″N 7°21′12.761″E	2255726	505300, 505400
11	Lac de Moiry	VS	2248	46°07′44.768″N 7°34′14.213″E	1309592	503200
12	Stausee Mattmark	VS	2195	46°02′16.908″N 7°57′36.736″E	1741439	501500, 501600
13	Lac de Salanfe	VS	1908	46°08′26.565″N 6°57′30.465″E	1795618	507700, 507600, 507500
14	Lac du Vieux Emosson	VS	2225	46°03′42.913″N 6°53′23.662″E	545561	506900, 506800, 506850, 506700
15	Lac d'Emosson	VS	1920	46°04′46.590″N 6°55′03.403″E	3214380	506900, 506800, 506850, 506700
16	Lac des Toules	VS	1809	45°55′08.903″N 7°11′55.159″E	599167	505900
17	Gigerwaldsee	SG	1331	46°54′52.718″N 9°22′24.901″E	689511	104700, 104600
18	Limmerensee	GL	1855	46°50′06.476″N 9°00′52.678″E	1340348	400100, 400200, 400250, 400400, 400050
19	Muttsee	GL	2474	46°51′49.489″N 9°01′41.577″E	410167	400100, 400050
20	Lag da Pigniu	GR	1447	46°49′48.710″N 9°06′12.977″E	390001	101000
21	Lai da Marmorera	GR	1676	46°30′02.559″N 9°38′09.970″E	1369543	103100, 103200
22	Lägh da l'Albigna	GR	2162	46°19′47.530″N 9°38′51.736″E	1258106	701200, 701000, 701100, 700800, 701400
23	Lai dad Ova spin	GR	1630	46°40′08.709″N 10°09′32.022″E	351190	800800
24	Lac de l'Hongrin	VD	1250	46°25′27.784″N 7°03′03.084″E	1552993	509100
25	Lago Dei Cavagnöö	TI	2310	46°27′16.427″N 8°30′05.141″E	476416	602500
26	Lago di Robièi	TI	1940	46°26′42.852″N 8°31′00.384″E	246134	602600, 602500
27	Lago del Zött	TI	1940	46°25′59.078″N 8°30′10.549″E	148255	602600
28	Lago Del Naret	TI	2310	46°28′38.474″N 8°34′08.384″E	731588	602500
29	Lago Del Sambuco	TI	1461	46°27′48.400″N 8°38′59.169″E	1109119	602400
30	Mattenalpsee	BE	1874	46°37′49.606″N 8°14′04.222″E	186439	200500
31	Lac Supérieur de Fully	VS	2130	46°10′42.715″N 7°05′38.072″E	203904	505600
32	Lagh da Palü	GR	1923	46°22′23.285″N 10°01′31.567″E	53615	700100, 700300
33	Lac de Cleuson	VS	2179	46°06′27.489″N 7°19′22.793″E	478894	504300
34	Lago della Sella	TI	2256	46°33′48.593″N 8°35′54.343″E	450778	600050, 600200, 600100
35	Griessee	VS	2386	46°27′27.473″N 8°22′14.130″E	636908	500100
36	[Löbbia unnamed]	GR	1416	46°22′35.312″N 9°39′29.383″E	29232	701200, 701000, 701100, 700800
37	[Palü unnamed]	GR	1923	46°22′18.512″N 10°01′27.150″E	3196	700100, 700300
38	[Ferpècle unnamed]	VS	1891	46°03′28.271″N 7°33′01.451″E	11174	504800
39	Zervreilasee	GR	1857	46°34′13.045″N 9°06′13.332″E	1564848	101100
40	[Fionnay unnamed 1]	VS	1484	46°02′02.917″N 7°18′17.297″E	29255	505300, 505000
41	[Fionnay unnamed 2]	VS	1491	46°02′00.911″N 7°18′31.832″E	17474	505300, 505000
42	Bortelsee	VS	2464	46°17′15.158″N 8°06′28.216″E	136491	501375, 501350
43	[Pallazuit unnamed]	VS	1327	45°58′54.120″N 7°11′20.977″E	15866	505900
44	Lai da Curnera	GR	1955	46°37′37.989″N 8°42′47.885″E	793864	100200
45	Lai da Nalps	GR	1904	46°37′53.009″N 8°45′47.373″E	901199	100200
46	Lai da Sontga Maria	GR	1906	46°34′32.128″N 8°47′43.351″E	1773494	100200
47	[Safien Platz unnamed 1]	GR	1294	46°40′47.942″N 9°19′00.192″E	29814	101200
48	Rabiusa	GR	1147	46°43′53.721″N 9°20′18.153″E	38051	101200
49	[Safien Platz unnamed 2]	GR	1720	46°36′56.418″N 9°16′42.940″E	36511	101200
50	Sanetschsee	VS	2033	46°21′24.504″N 7°17′39.240″E	284234	203600
51	Lago d'Isola	GR	1602	46°26′59.160″N 9°11′16.088″E	363253	601400
52	[Spina (Isola) unnamed]	GR	1191	46°25′43.141″N 9°12′27.602″E	25958	601400
53	Arnesee	BE	1541	46°23′20.605″N 7°13′01.871″E	449916	508700
54	Lago di Lucendro	TI	2134	46°33′43.358″N 8°32′27.926″E	542675	600100
55	[Airolo unnamed]	TI	1129	46°31′33.916″N 8°36′16.108″E	47827	600100
56	[Pradella unnamed]	GR	1141	46°48′14.232″N 10°20′06.790″E	33245	801000
57	[Châtelard-Vallorcine unnamed 1]	VS	1119	46°03′07.796″N 6°57′02.065″E	10822	507100, 506700
58	[Châtelard-Vallorcine unnamed 2]	VS	1515	46°02′53.915″N 6°57′26.714″E	24012	507100, 506700
59	[Vissoie unnamed 1]	VS	1119	46°12′42.622″N 7°35′06.243″E	11086	503300
60	[Vissoie unnamed 2]	VS	1560	46°09′09.184″N 7°37′16.028″E	22469	503300, 503200
61	Oberer Murgsee	SG	1819	47°02′21.174″N 9°09′14.903″E	196690	403000
62	Göscheneralpsee	UR	1792	46°38′43.280″N 8°29′00.990″E	1304567	300400
63	Sufnersee	GR	1398	46°33′59.088″N 9°22′08.944″E	823086	102100
64	Lai da Seara	GR	1080	46°35′14.964″N 9°25′15.253″E	67821	102100
65	Melchsee	OW	1891	46°46′14.847″N 8°16′20.967″E	507012	303800, 303650
66	Tannensee	OW	1976	46°46′25.190″N 8°18′23.212″E	339608	303800, 303650
67	Lago di Luzzone	TI	1606	46°34′00.390″N 8°58′36.032″E	1401350	601200
68	Lago Ritóm	TI	1850	46°32′26.225″N 8°41′25.633″E	1461650	600400
69	Lac de Tseuxier	VS	1774	46°21′01.878″N 7°25′50.353″E	829589	503500, 503700
70	Bannalpsee	NW	1586	46°52′04.059″N 8°25′47.114″E	157598	303300

Table S3B. Full water body sample and collected attributes, Related to STAR Methods. *: Data for associated hydro installations can be found in (Swiss Federal Office of Energy, 2019).

Site ID	Name	Canton	Altitude (m)	Coordinates (WGS 84)	Surface Area (m²)	Associated Hydro Installations (ZE-Nr.) *
71	Lai da Burvagn	GR	1116	46°37′16.944″N 9°35′13.116″E	47529	103200
72	Lag da Breil	GR	1254	46°46′17.363″N 9°04′15.743″E	62743	100300, 101000
73	Stausee Gibidum	VS	1436	46°22′26.493″N 8°00′11.565″E	204456	501200
74	Turtmannsee	VS	2176	46°10′07.068″N 7°41′34.562″E	98406	502800, 503200, 502600
75	Stausee Garichti	GL	1622	46°57′20.532″N 9°05′58.445″E	152984	401500
76	Illsee	VS	2359	46°15′25.790″N 7°37′54.752″E	208876	502700, 502600
77	Oberer Märetschisee	VS	2360	46°15′19.194″N 7°39′01.867″E	42441	502700, 502600
78	Unterer Märetschisee	VS	2305	46°15′28.413″N 7°39′15.922″E	43309	502700, 502600
79	[Zermeiggern unnamed]	VS	1738	46°04′57.470″N 7°57′26.917″E	14630	501500, 501600
80	[Oberems (Argessa) unnamed]	VS	1370	46°16′58.001″N 7°41′23.306″E	3465	502600
81	[Châtelard-Barberine unnamed]	VS	1115	46°03′40.544″N 6°57′33.964″E	17142	506700
82	[Peccia (Sambuco) unnamed]	TI	1032	46°24′50.832″N 8°36′37.300″E	17408	602400

Table S4. Outline of investigated floating solar design configurations, Related to Section 3.4. Optimizing for winter production versus total production may result in different azimuth angles.

Panel Configuration	Description
Case 1: Flat panels	Panels placed horizontally on surface
Case 2: Tracking panels	Panel normal vector is aligned with solar position at all times
Case 3: Fixed panels with 12-degree tilt, optimized for total production	Total output optimization of standard product from current floating solar market leader (Ciel & Terre International, 2019)
Case 4: Fixed panels with 12-degree tilt, optimized for winter production	Winter output optimization of standard product from current floating solar market leader (Ciel & Terre International, 2019)
Case 5: Fixed panels with tilt between 30 and 65 degrees, optimized for winter production	Configuration to maximize winter production with high-altitude fixed panels (Kahl et al., 2019)

Table S5. Retrieved values for floating solar capital costs, Related to Table 1.

Source	Floating Solar Capital Cost	Value Used for Average (CHF/Wp)
(World Bank Group, 2019)	Range = 0.8 to 1.2 USD/Wp	0.99 (center of range)
(Campana et al., 2019)	2.35 USD/Wp	2.33
(Silvério et al., 2018)	3.72 BRL/Wp	0.97

Table S6. Exchange rates used in our costs analysis, Related to Table 1. *: Source = OANDA Currency Converter (OANDA, 2019) – retrieved on August 3rd, 2019.

Currency	Equivalent CHF Rate *
USD	0.99
EUR	1.09
BRL	0.26

Table S7. Top 10 sites in our sample ranked by total expected annual production. Lac d'Emosson and Lac de Salanfe are highlighted as the only 2 sites among the top 10 for both total output and output per square meter, Related to Section 4.

Total Output Potential Rank	Water Body	Altitude (m)	Surface Area Rank	Output per Square Meter Rank	Annual Flat Output per Square Meter (kWh)	Annual Flat Output at 10% Coverage (GWh)
1	Lac des Dix	2362	1	54	170	67.9
2	Lac d'Emosson	1920	2	6	196	62.9
3	Grimselsee	1909	3	55	170	45.6
4	Lac de Mauvoisin	1969	4	47	175	39.6
5	Lac de Salanfe	1908	5	4	199	35.7
6	Oberaarsee	2303	8	23	187	30.4
7	Lac de l'Hongrin	1250	10	17	191	29.7
8	Lai da Songta Maria	1906	6	67	165	29.3
9	Stausee Mattmark	2195	7	69	163	28.4
10	Lago Ritóm	1850	11	25	185	27.1

Table S8. Top 10 sites in our sample ranked by economic viability (equivalent to lowest LCOE and highest output per square meter). Lac d'Emosson and Lac de Salanfe are highlighted as the only 2 sites among the top 10 for both technical potential and economic viability, Related to Section 4. Note: Locations of unnamed water bodies can be found in **Table S3**.

Economic Viability Rank	Water Body	Altitude (m)	Surface Area Rank	Annual Flat Output per Square Meter (kWh)	Annual Flat Output at 10% Coverage (GWh)
1	Lac Supérieur de Fully	2130	46	210	4.3
2	Stausee Gibidum	1436	45	209	4.3
3	[Vissoie unnamed 1]	1119	79	202	0.2
4	Lac de Salanfe	1908	5	199	35.7
5	Zmuttbach	1968	63	197	0.8
6	Lac d'Emosson	1920	2	196	62.9
7	[Spina (Isola) unnamed]	1191	70	193	0.5
8	[Peccia (Sambuco) unnamed]	1032	74	192	0.3
9	[Châtelard-Barberine unnamed]	1115	75	192	0.3
10	[Châtelard-Vallorcine unnamed1]	1119	80	192	0.2

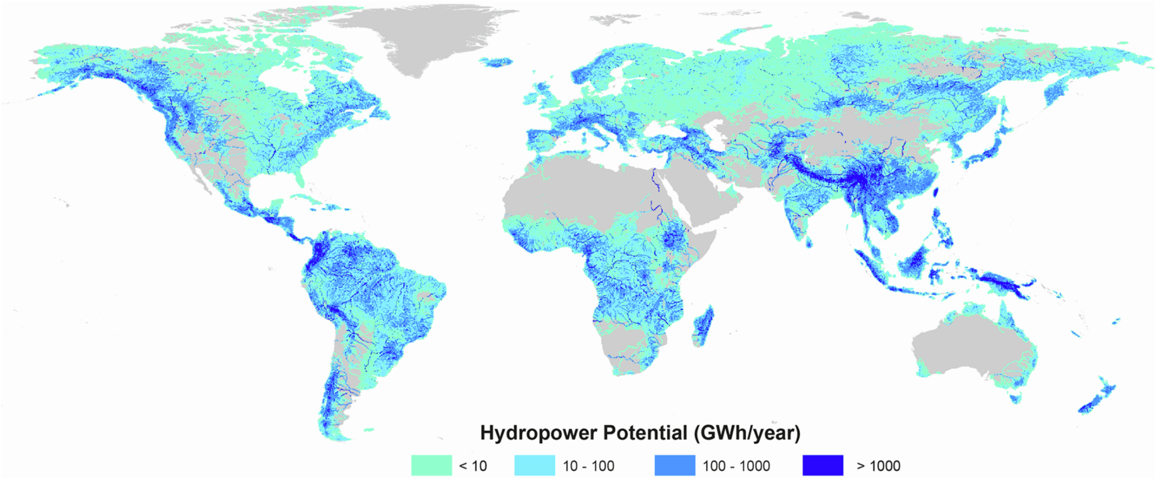

Figure S1. Global map of hydropower potential. Sourced from (Hoes et al., 2017), Related to STAR Methods.

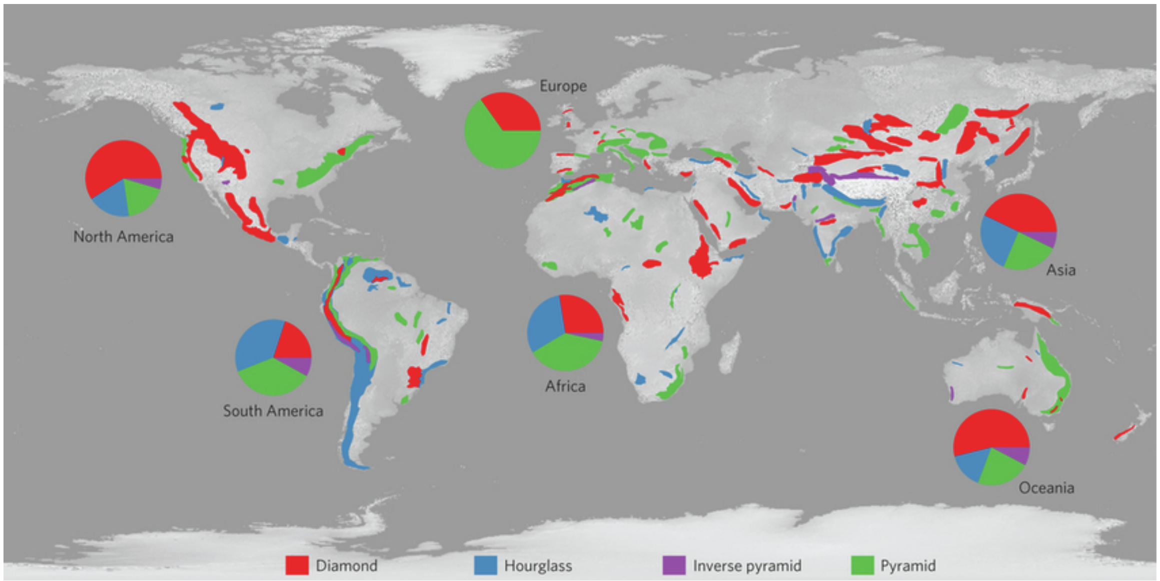

Figure S2. Global map of mountain ranges and types, Related to STAR Methods. Sourced from (Elsen et al., 2015).

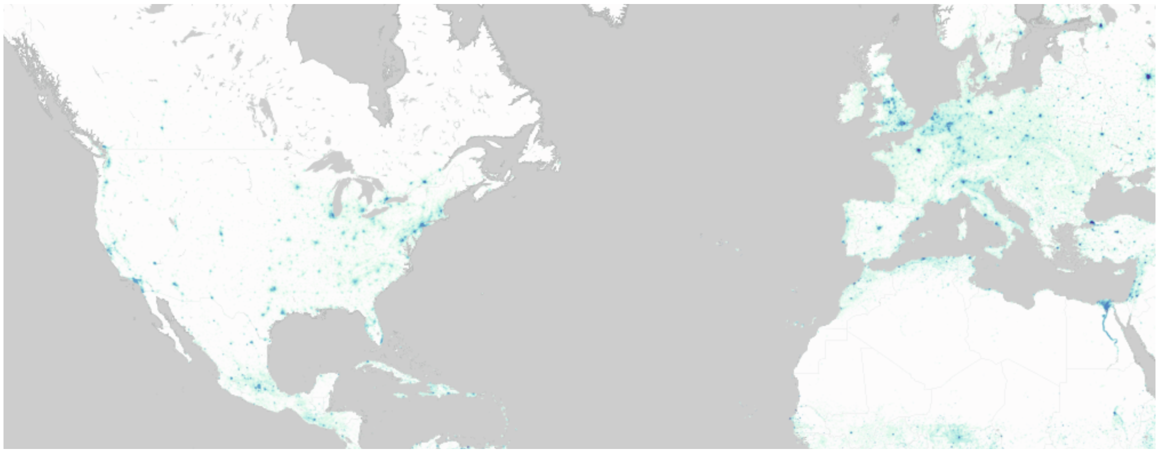

Figure S3. Snippet from visual data set of global population density, Related to STAR Methods. Sourced from (Schiavina et al., 2020).
